# Unraveling the adaptive strategies of *Mycoplasma hominis* through proteogenomic profiling of clinical isolates

**DOI:** 10.3389/fcimb.2024.1398706

**Published:** 2024-05-02

**Authors:** Olga V. Pobeguts, Maria A. Galaymina, Kirill V. Sikamov, Diana R. Urazaeva, Alexander S. Avshalumov, Maria V. Mikhailycheva, Vlad V. Babenko, Igor P. Smirnov, Alexey Yu. Gorbachev

**Affiliations:** Department of Molecular Biology and Genetics, Federal State Budgetary Institution Lopukhin Federal Research and Clinical Center of Physical-chemical Medicine Federal Medical Biological Agency, Moscow, Russia

**Keywords:** *Mycoplasma hominis*, clinical isolates, mini colonies, phenotype switch, proteogenomic analysis, adaptation to host

## Abstract

**Introduction:**

*Mycoplasma hominis* (*M. hominis*) belongs to the class *Mollicutes*, characterized by a very small genome size, reduction of metabolic pathways, including transcription factors, and the absence of a cell wall. Despite this, they adapt well not only to specific niches within the host organism but can also spread throughout the body, colonizing various organs and tissues. The adaptation mechanisms of *M. hominis*, as well as their regulatory pathways, are poorly understood. It is known that, when adapting to adverse conditions, *Mycoplasmas* can undergo phenotypic switches that may persist for several generations.

**Methods:**

To investigate the adaptive properties of *M. hominis* related to survival in the host, we conducted a comparative phenotypic and proteogenomic analysis of eight clinical isolates of *M. hominis* obtained from patients with urogenital infections and the laboratory strain H-34.

**Results:**

We have shown that clinical isolates differ in phenotypic features from the laboratory strain, form biofilms more effectively and show resistance to ofloxacin. The comparative proteogenomic analysis revealed that, unlike the laboratory strain, the clinical isolates possess several features related to stress survival: they switch carbon metabolism, activating the energetically least advantageous pathway of nucleoside utilization, which allows slowing down cellular processes and transitioning to a starvation state; they reconfigure the repertoire of membrane proteins; they have integrative conjugative elements in their genomes, which are key mediators of horizontal gene transfer. The upregulation of the methylating subunit of the restriction-modification (RM) system type I and the additional components of RM systems found in clinical isolates suggest that DNA methylation may play a role in regulating the adaptation mechanisms of *M. hominis* in the host organism. It has been shown that based on the proteogenomic profile, namely the genome sequence, protein content, composition of the RM systems and additional subunits HsdM, HsdS and HsdR, composition and number of transposable elements, as well as the sequence of the main variable antigen Vaa, we can divide clinical isolates into two phenotypes: typical colonies (TC), which have a high growth rate, and atypical (aTC) mini-colonies, which have a slow growth rate and exhibit properties similar to persisters.

**Discussion:**

We believe that the key mechanism of adaptation of *M. hominis* in the host is phenotypic restructuring, leading to a slowing down cellular processes and the formation of small atypical colonies. This is due to a switch in carbon metabolism and activation the pathway of nucleoside utilization. We hypothesize that DNA methylation may play a role in regulating this switch.

## 1 Introduction

*Mycoplasma hominis* (*M. hominis*) belongs to the class *Mollicutes*, characterized by the absence of a cell wall, reduced genome size (about 600 protein-encoding genes), and consequently, a reduction in metabolic pathways. It is considered an opportunistic human pathogen capable of causing acute and chronic infections of the urogenital tract (Waites and Talkington, 2005). Intrauterine infection by this *Mycoplasma* can lead to meningitis, pneumonia, and abscesses in newborns (Waites and Talkington, 2005). There are known cases of acute and chronic pyelonephritis caused by *M. hominis* (Wong and Yuen, 1995). It has been shown that these *Mycoplasmas* can adhere to blood cells and thus spread throughout the organs and tissues, causing a generalized mycoplasmal infection (Rakovskaya et al., 2013). Another feature of this bacterium is the long-term persistence of the pathogen in the tissues of the infected organism (Baseman and Tully, 1997). It is assumed that *M. hominis* residing in the human urogenital tract has oncogenic potential and promotes the immortalization, increased migration, and invasion of tumor cells (Huang et al., 2001).

Despite the reduced genome, very small cell size, and absence of a cell wall, *M. hominis* possesses a high adaptive potential, allowing these bacteria to evade the action of the host’s immune system and cause chronic inflammation without obvious clinical symptoms. Previously, it was believed that *Mycoplasmas* are strictly associated with a specific niche in the organism, but it is now known that mycoplasmas can spread throughout the organs and tissues. One of the adaptive mechanisms of mycoplasmas is adhesion and intracellular invasion. Many *Mycoplasmas* are capable not only of attaching to the host’s eukaryotic cells but also of penetrating these cells, freely replicating within them, modulating cellular apoptosis without causing cell death (Razin et al., 1998; Gerlic et al., 2007; Zuo et al., 2009; Kornspan et al., 2010). It has been shown that adaptation of *Mycoplasmas* during infection of eukaryotic cells is associated with phenotypic switching, which can persist for a long time over several generations and is associated with the restructuring of metabolic pathways (Matyushkina et al., 2016; Fisunov et al., 2022). *Mycoplasmas* can also activate a pro-inflammatory response and bind immunoglobulins, thereby inactivating their functions (Grover et al., 2014; Arfi et al., 2016). Another mechanism is phenotypic plasticity, caused by constant changes in surface antigens, allowing them to evade the immune system (van der Woude and Bäumler, 2004; Citti et al., 2010). However, the molecular bases and regulation of such adaptation are poorly understood. *Mycoplasmas* have a very limited set of transcriptional regulators (5-10 known gene expression regulators per *Mycoplasma* genome). It is assumed that gene expression in *Mycoplasmas* is maintained and coordinated not so much by promoter activity but by other, as yet unknown mechanisms (Mazin et al., 2014). Most studies on the adaptation mechanisms of *M. hominis* have been conducted on laboratory strains, which were isolated from the host organism too long ago and adapted to optimal cultivation conditions in rich media. In this work, we decided to study clinical isolates of *M. hominis* obtained from patients with urogenital infections.

*Mycoplasmas* can retain a phenotype adapted to life inside the host over several generations. Therefore, we conducted a comprehensive study of clinical isolates at the genome and proteome level which allowed us to understand: how they differ from the laboratory strain, what they have in common, how they differ from each other and whether there are universal mechanisms for adaptation or each isolate has unique ways to survive and resist adverse conditions.

## 2 Materials and methods

### 2.1 Bacterial strain and growth conditions

*M. hominis strain* H-34 was kindly provided by Dr. K.H. Lemke (Lister Institute of Preventive Medicine, London, UK). Clinical isolates of *M. hominis* (MHO43, MHO7, MHO11, MHO40, MHO33, MHO12, MHO1862, MHO45), obtained from patients with urogenital infections, were provided by Ph.D. Taraskina A.N. (Research Institute named after the Department of Obstetrics, Gynecology and Reproductology, St Petersburg, Russia). *M. hominis* strain H-34 (MHOH34) and clinical isolates were cultivated on Brain Heart Infusion medium (BHI) supplemented with 15% horse serum (Gibco, Thermo Fisher Scientific, USA), 5% yeast extract and 1% L-arginine as the sole carbon source. Penicillin was added up to 100 µg/ml^-1^ to avoid contamination. Solid agar (1.3%), supplemented with 15% horse serum, 5% yeast extract, 1% L-arginine and penicillin was used to analyze the morphology and size of colonies formed by clinical isolates and the laboratory strain. Colonies that appeared on BHI agar plates were observed with the light microscope with a 40X objective (LETZLAR, Germany). The increase in the DNA level was used to measure growth curves. One ml of culture was taken for DNA extraction at each time point. All DNA quantity values are the average of triplicate experiments with standard deviations.

### 2.2 Antibiotic test

Since *M. hominis* form very small colonies on solid agar we decided to conduct comparative analysis based on the amount of DNA, obtained from cell culture in the presence or absence of 5 µg/ml ofloxacin. The laboratory strain *M. hominis* MHOH34 and clinical isolates were cultured on a brain-heart infusion medium (BHI) with the addition of 15% horse serum (Gibco, Thermo Fisher Scientific, USA), 5% yeast extract, and 1% L-arginine in the presence of 5 μg/ml ofloxacin and its absence control.

### 2.3 Plate assay for biofilm quantification

Biofilm density was measured using a crystal violet assay as described in (McAuliffe et al., 2006). The laboratory strain *M. hominis* MHOH34 and clinical isolates were cultured in 96-well plates for 7 days. The optical density was measured in the plate at OD600 nm. The wells in the plate were washed 2 times with PBS to remove non-adherent cells and stained with 0.5% crystal violet solution for 30 minutes. The wells were then washed five times in distilled water and dried at room temperature. 200 μl of 96% ethanol was added to the wells and incubated for 30 minutes. Biofilm formation was quantified by measuring absorption at OD550 nm. The biofilm formation index was considered as a ratio of OD550 to OD600 nm.

### 2.4 DNA isolation

Genomic DNA extraction was performed using the Invitrogen PureLink Genomic Mini Kit according to the manufacturer’s instructions using a bacterial cell lysate protocol without lysozyme treatment. Mycoplasma cells were washed three times with 50 mM Tris-HCl buffer (pH 7.4) containing 150 mM NaCl and 5 mM MgCl_2_ before DNA extraction to eliminate nucleic acids from the medium.

### 2.5 Genome sequencing, assembly, and annotation

**Figure 1:**
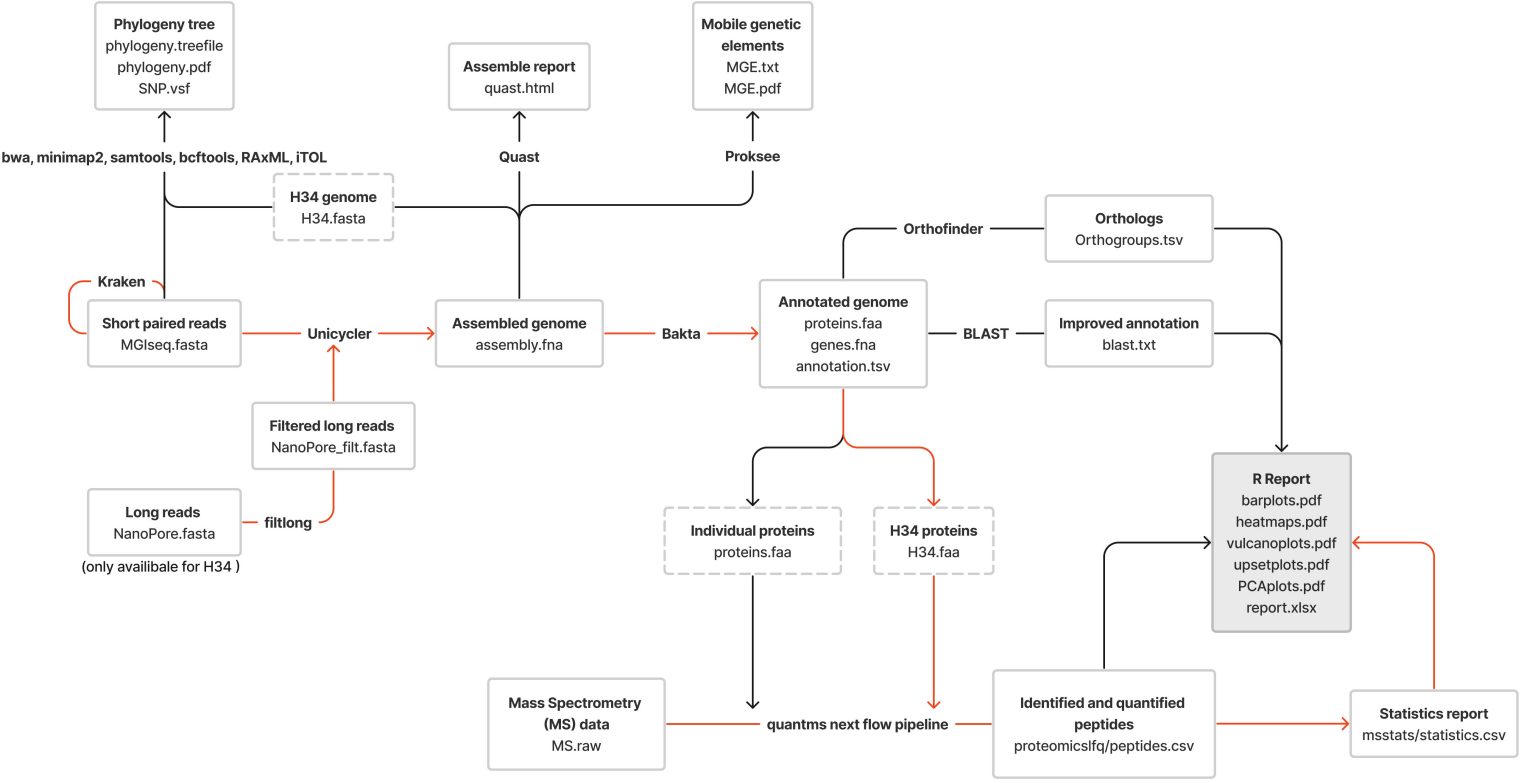
The pipeline used for the proteogenomic profiling of clinical isolates of *M. hominis*; the red lines represent the proteomic profiling route; the black lines show the genomic profiling route.

To prepare DNA libraries for sequencing, MGIEasy Universal DNA Library Prep Set and the “User Manual” protocol (Cat. No.: 1000006985, 1000006986, 1000017571. Set Version: V1.0. Manual Version: A3), were used (https://en.mgi-tech.com/products/reagents_info/8/). The protocol includes the following steps: DNA fragmentation, selection of DNA fragments by length, end repair and A-tailing, adapter ligation, post-ligation purification, PCR, post-PCR library purification, denaturation, single-strand circularization and product purification. To perform sequencing from ready-made DNA libraries, protocol DNBSEQ-G400RS High-throughput Rapid Sequencing Set User Manual A2 was used. Sequencing was performed on a DNBSEQ-G400RS MGITECH sequencer and Nanopore sequencer PromethlON (Oxford Nanopore Technologies). The pipeline used for proteogenomic profiling of *M. hominis* clinical isolates is shown in **Figure 1**. Quality control and filtering of reads were performed using FastQC and trimmomatic for short MGISEQ reads (150 nt) and filtlong for long Oxford NanoPore reads. Taxonomic validation through the Kraken program confirmed the affiliation of sequenced reads to *M. hominis.* Genome assembly for isolates relied on Unicycler Assembly, utilizing only short reads. The assembly of the laboratory strain MHOH34 involved hybrid assembly incorporating both short and long reads, with the “bold” mode specified to assemble the MHOH34 reference genome into a single contig (circular structure).

Assembly quality control was executed using the Quast program. Genome annotation was performed using Bakta, incorporating parameters characteristic of *M. hominis* (specifically, translation-table - 4). The protein sequences of the well-annotated ATCC 23114 strain downloaded from NCBI (GCF_000085865.1) were used as a reference protein database for annotation refinement. For improved annotation and identification of specific genes/proteins of interest, the BLAST program was employed with complete nucleotide and protein databases downloaded from NCBI. Orthologous protein search among isolates utilized Orthofinder software. The genome sequences of the laboratory strain H-34 and 8 clinical isolates of *M. hominis* have been deposited at NCBI under the accession numbers BioProject ID: PRJNA1067906.

### 2.6 Identification of SNP variants and phylogenetic tree construction

The phylogenetic tree was constructed based on SNP identification between isolates, with reads aligned to the MHOH34 reference genome using alignment tools such as bwa and minimap2. Post-processing of alignment files involved Samtools, SNP calling utilized BCFtools, and the phylogenetic tree was constructed using RAxML. Visualization of the phylogenetic tree was achieved through iTOL.

### 2.7 Annotation of mobile elements

Annotation of mobile elements in the genome utilized the online tool Proksee described in (Grant et al., 2023). In addition to this, genome annotation was used using BLAST and Bakta.

### 2.8 Free-gel digestion of protein samples

Clinical isolates reached the logarithmic growth phase at different times, so for proteomic analysis they were cultured for different times based on growth curves. In addition, different amounts of bacterial culture were taken for proteomic analysis. For isolates MHOH34 and MHO12 - 5 ml of culture, for isolates MHO33, MHO40, MHO43, MHO45 and MHO7 - 10 ml of culture, for isolates MHO1862 and MHO11 - 15 ml of culture. Cells were washed with 50 mM Tris-HCl buffer (pH 7.4) containing 150 mM NaCl and 5 mM MgCl_2_ (buffer A) twice. Further, 10 μL of 10% sodium deoxycholate (DCNa) and 0.5 μL nuclease mix (GE Healthcare) were added to the cell pellet. After incubation at 4°C for 1 h, the samples were resuspended in 100 µL of 100 mM Tris-HCl buffer (pH 8.5) containing 0.1% DCNa, 8 M urea, and 2.5 mM EDTA After incubation for 20 min, samples were centrifuged at 14,000 g at 4°C for 10 min to remove intact cells and debris. The supernatant was collected, and the protein concentration was measured using a Bradford Assay Kit (BioRad). Disulfide bonds were reduced in the supernatant containing 100 μg of total protein by the addition of Tris(2-carboxyethyl) phosphine hydrochloride (TCEP) (Sigma) to a final concentration of 5 mM, and the reaction was incubated at 37°C for 60 min. To alkylate-free cysteines, chloroacetamide (BioRad) was added to a final concentration of 30 mM and the solution was placed at room temperature (RT) in the dark for 30 min. The TCEP addition was repeated. The sample was diluted six-fold with 50 mM Tris-HCl (pH 8.5), containing 0.01% DCNa and Trypsin (Trypsin Gold, Mass Spectrometry Grade, Promega) to achieve a final trypsin: protein ratio of 1:50 (w/w), and it was subsequently incubated at 37°C overnight. To stop trypsinolysis and to degrade the acid-labile DCNa, trifluoroacetic acid (TFA) was added to a final concentration of 0.5% (v/v) (the pH should be less than 2.0), and the samples were incubated at 37 °C for 45 min. Further, the samples were centrifuged at 14,000 g for 10 min to remove DCNa. The peptide extract was desalted using a Discovery DSC-18 tube (Supelco) according to the manufacturer’s protocol. Peptides were eluted with 1 mL of 75% acetonitrile (ACN) solution containing 0.1% TFA, dried in a SpeedVac (Labconco), and resuspended in a 3% ACN solution containing 0.1% TFA to a final concentration of 5 μg/μL. Samples of TCs and MCs were subjected to the same degree of washing and digestion.

### 2.9 LC-MS analysis

The LC-MS analysis of proteome samples was performed using Orbitrap Q Exactive HF-X (Thermo Fisher Scientific, Waltham, MA) mass spectrometer. For ionization, a nano-electrospray (nano-ESI) source was used in conjunction with high-pressure nanoflow chromatography UPLC Ultimate 3000 (Thermo Fisher Scientific, Waltham, MA). The lab-made reverse-phase column (ID 100mm with length 500mm of fused silica TSP100375 (Molex, Lisle, IL) was packed with phase Kinetex C18, 2.4 μm (Phenomenex, Torrance, CA) using pressure injection cell (Next Advance, Troy, NY). During HPLC run it was thermostatically controlled at 60°C. Samples were loaded in buffer A (0.1% Formic acid) and eluted with a linear (90 min) gradient of 3 to 55% buffer B (0.1% Formic acid, 80% Acetonitrile) at a flow rate of 220 nl/min. Mass spectrometric data were stored during automatic switching between MS1 scans and up to 12 MS/MS scans (TopN method). The target AGC value for MS1 scanning was set to 3x10e6 in the range 390-1400 m/z with a maximum ion injection time of 45 ms and resolution of 60000. The precursor ions were isolated at a window width of 2.0 m/z and then were fragmented by high-energy dissociation with a normalized collision energy of 30 eV. MS/MS scans were saved with a resolution of 30000 at 400 m/z and AGC of 10e5 for target ions with a maximum ion injection time of 50 ms. Data is available via ProteomeXchange with the identifier PXD048835 (DOI:10.6019/PXD048835).

### 2.10 Protein identification, quantification, and comparative proteomic profiling

The Nextflow pipeline QuatMS (10.5281/zenodo.7754148) was employed for processing mass spectrometry proteomic data, encompassing quantitative protein determination and identification. The protein database file used for this analysis consisted of protein sequences from the assembled and annotated H-34 reference. Post-processing of the peptide intensity file, including normalization and statistical analysis, was carried out using the R programming language and the functionality of the MSstats library. Graph construction and post-processing of obtained data utilized an R script, accessible via GitHub (https://github.com/Sikamov/M.hominis_2023).

## 3 Results and discussion

### 3.1 Phenotypic characteristics of clinical isolates

**Figure 2:**
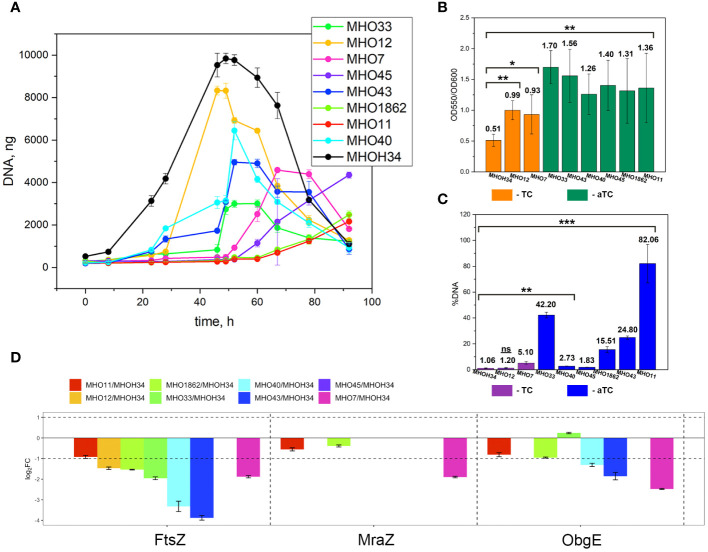
**(A)** growth curves based on the amount of DNA of laboratory strain H-34 (MHOH34) and eight clinical isolates of *M. hominis* (MHO12, MHO7, MHO33, MHO40, MHO43, MHO45, MHO11, MHO1862), cultivated in modified BHI medium. One ml of culture was taken for DNA extraction at each time point. All DNA quantity values are the average of triplicate experiments with standard deviations. **(B)** the ability of laboratory strain MHOH34 and eight clinic isolates of *M. hominis* to form biofilms. Biofilm production was quantified by measuring the absorbance (550 nm) of crystal violet in a microplate. Statistical significance is indicated (*p<0.001, **p<0.0001, one-way ANOVA). **(С)** susceptibility of laboratory strain MHOH-34 and eight clinic isolates of *M. hominis* to ofloxacin. The assay is based on the amount of DNA obtained from cell culture in the presence or absence of 5 μg/ml ofloxacin. Differences between DNA values for antibiotic-treated samples and untreated positive control samples are shown. Data are represented as mean ± SEM. Statistical significance is indicated (*p<0.001, **p<0.0001, one-way ANOVA). **(D)** change in the abundance of proteins FtsZ, MraZ and ObgG associated with cell division obtained by LC-MS analysis for the laboratory strain MHOH34 and in clinical isolates of *M. hominis*.

**Figure 3:**
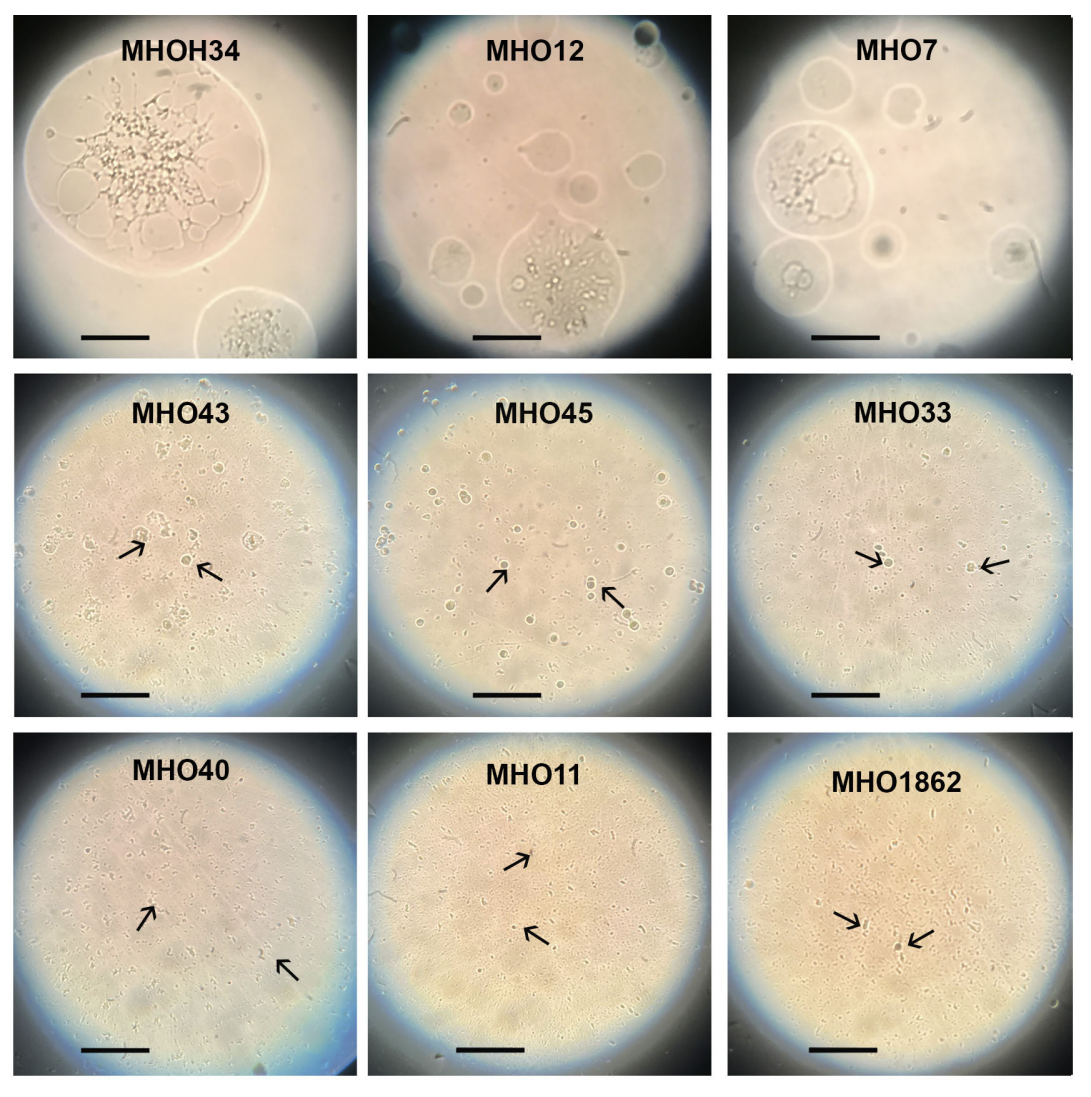
Phenotype of colonies (the laboratory strain MHOH34 and in clinical isolates of *M. hominis* (MHO12, MHO7, MHO33, MHO40, MHO43, MHO45, MHO11, MHO1862). Colonies were observed with the light microscope with the 40x objective (LETZLAR, Germany). The laboratory strain MHOH34 and clinic isolates MHO12, MHO7 form typical colonies (TCs) of about 250-350 µm. Clinic isolates MHO33, MHO40, MHO43, MHO45, MHO11, MHO1862 form atypical colonies (aTCs) in size less than 30 µm. Arrows indicate mini colonies (aTCs) of *M. hominis*. The black bar represents 100 µm.

Comparative analysis of growth rates showed that all clinical isolates exhibited a decrease in growth rate compared to the laboratory strain MHOH34 but to varying degrees (**Figure 2A**). **Table 1** presents the average values of the maximum amount of DNA reached by each isolate when grown in a medium with the addition of arginine. The table also indicates the time at which these maximum values are reached. The growth intensities of isolates MHO12 and MHOH34 are almost identical, while the other isolates grow more slowly, with isolates MHO1862 and MHO11 showing the lowest growth rate. To determine the size and morphology of colonies, all *M. hominis* strains were grown on agar plates (**Figure 3**). It turned out that unlike the laboratory strain, which is characterized by round colonies of about 250-350 µm, clinical isolates form colonies of varying sizes. Phenotypically, we divided them into two groups: isolates that form typical colonies, similar in size and morphology to the laboratory strain (MHO12, MHO7 - TCs) and isolates (MHO33, MHO43, MHO45, MHO40, MHO11, and MHO1862) that form mini-colonies, much smaller in size (less than 30 µm), which we called atypical colonies (aTCs). Such phenotypic differences in colonies are already known for *Mycoplasmas* (Rakovskaya et al., 2019). The atypical colonies were first discovered by I.V. Rakovskaya and colleagues when culturing blood serum from patients with various inflammatory diseases on agar plates with selective media for *Mycoplasmas*. They differed from TCs in shape, size, extraordinary resistance to adverse factors, and ability to grow without arginine in the culture medium without arginine in the culture medium. Similar aTCs were also obtained *in vitro* under the influence of adverse factors (treatment with antibodies, various types of non-thermal plasma, cultivation in a poor medium, or prolonged incubation) on a *Mycoplasma* culture. The authors suggested that in the host infected with *M. hominis*, there exists a phenotype resistant to adverse factors, capable of hiding from the immune system and freely persisting inside the host. We previously showed that the formation of aTCs is associated with the restructuring of energy metabolism, contributing to the formation of a persisting phenotype [11]. We also previously observed changes in colony size in an infectious model of HeLa cell infection with *M. hominis*. It was found that before infection, *M. hominis* cells formed TCs, and after acute infection of HeLa, we observed a decrease in colony size and the appearance of aTCs (Galyamina et al., 2022). Biofilm quantification analysis (**Figure 2B**) showed that all strains are capable of forming biofilms, with the laboratory strain having the lowest ability, with an average OD600/OD550 value of 0.51. Apart from isolates MHO12 and MHO7, all clinical isolates have an OD600/OD550 value exceeding 1. We noticed that isolates forming TCs (MHOH34, MHO12, MHO7) have a lower ability to form biofilms than isolates forming aTCs. An antibiotic test was performed using ofloxacin, which inhibits DNA replication by affecting the activity of DNA gyrase and topoisomerase IV and is a fluoroquinolone. It is known that *Mycoplasmas*, including *M. hominis*, are sensitive to fluoroquinolones (Bébéar et al., 2000), which, according to clinical recommendations, are effectively used in the treatment of *Mycoplasma* infections. In addition, we previously showed that the laboratory strain MHOH34 has significant sensitivity to ofloxacin (Evsyutina et al., 2022; Fisunov et al., 2022). Since it is difficult to count very small colonies on solid agar, we conducted a comparative analysis based on of the amount of DNA obtained from cell culture isolates with the addition of 5 µg/ml ofloxacin relative to the control without antibiotic (**Figure 2C**). The isolates exhibit different sensitivity to the antibiotic. The most sensitive were the laboratory strain MHOH34 and isolate MHO12 (1.06% and 1.2% of DNA remained after culture growth in the presence of ofloxacin compared to the control). The other isolates were more resistant to the antibiotic, among them isolates MHO11, MHO43, MHO33, and MHO1862 (80,06%, 24,8%, 42,20% and 15,50% of DNA, respectively).

**Table 1:** Phenotypic characteristics of clinical isolates and laboratory strain MHOH34 of *M. hominis*, including the type of colonies (TCs- typical colonies, aTCs - atypical colonies), as well as the values of the maximum amount of DNA that isolates reach when growing on a BHI medium supplemented with arginine.

*M. hominis* isolates	maximum amount of the DNA, ng	growth time at which maximum amount of the DNA is reached, h	colonies type	pairwise comparisons with laboratory strain MHOH34 using ANOVA test, p-value
MHOH34	9767 ± 240.07	48	TС	–
MHO12	8333 ± 207.85	48	TС	5.6e-01
MHO7	4587 ± 100.17	67	ТС	3.2e-04
MHO33	2997 ± 165.02	52	aTС	1.1e-04
MHO43	4955 ± 129.87	52	аТС	9.7e-03
MHO45	4354 ± 144.04	92	аТС	6.0e-06
MHO1862	2485 ± 171.98	92	аТС	1.3e-06
MHO11	2163 ± 309.89	92	аТС	7.9e-07
MHO40	6448 ± 435.86	52	аТС	1.4e-02

### 3.2 Comparative analysis of the genomes of the laboratory strain MHOH34 and 8 clinical isolates

We determined that clinical isolates differ from the laboratory strain in growth rate and have had variations in colony phenotype. As a next step, we compared the genomes of the clinical isolates and the laboratory strain. Whole-genome sequencing of the clinical isolates on the DNBSEQ-G400RS MGITECH sequencer produced only short reads, assembled into 2 or 4 contigs. The general characteristics of the genomes are listed in **Table 2**. For the laboratory strain MHOH34, nanopore sequencing was also conducted on an Oxford Nanopore Technologies device to obtain long reads. As a result, the genome of MHOH34 was assembled into a circle. Taxonomy verification was performed using the KRAKEN program and Ribosomal Multilocus Sequence Typing (https://pubmed.ncbi.nlm.nih.gov/22282518/), and all isolates were classified into the class *Mollicutes*, genus *Mycoplasma*, and species *M. hominis*. The complete genome of the laboratory strain consists of one circular chromosome of 757,124 bp with a GC content of 26.8%. The genome was identified to have 609 protein-coding genes, with the coding portion of the genome accounting for 92.4%. All genomes showed a low GC content (~27%) and other genomic features typical for *M. hominis*. Among the clinical isolates, MHO1862 (727,136 bp) and MHO33 (715,039 bp) had the highest number of genome sequences resolved by the sequencing technology used, due to a higher number of transposable elements. Isolates MHO40, MHO45, MHO12, MHO11, and MHO43 were shown to have genome sequence sizes ranging from 681,052 to 701,387 bp. The smallest genome sequence size was detected in MHO7 (677,150 bp).

**Table 2:** General characteristics of the genomes of the laboratory strain MHOH34 and clinical isolates of *M. hominis*.

*M. hominis* strain	MHOН34	MHO7	MHO11	MHO12	MHO33	MHO40	MHO43	MHO45	MHO1862
Genome size(bp)	757124	677150	702003	681052	715039	710078	701387	696304	727136
N50^*^	757124	233519	47949	233519	130719	141561	47949	79758	266680
L50^*^	1	2	4	2	2	2	4	2	2
GC content	26.8	26.86	26.89	27.08	26.88	26.8	26.89	26.78	27.25
Protein-coding genes (excluding pseudogenes)	609	575	627	578	650	581	628	573	603
Gene/Genome (%)	92.4	92.5	92.1	92.5	92.5	92.7	92.2	92.8	92.4
Pseudogenes	5	6	3	6	1	4	5	3	2
tRNA number	37	33	33	33	34	34	33	33	34
rRNA (by *de novo* prediction)	6	4	4	5	4	4	4	4	4
hypothetical proteins	13	26	63	28	77	30	63	30	23
Short reads	1521082	1046706	707208	1600001	995735	1333539	912826	847862	1362894
Depth	441.43X	303.49X	183.92X	453.92X	233.67X	353.45X	235.18X	229.03X	346.88X
number of HTG	8	27	26	28	25	30	28	33	15

N50^*^- maximum contig length is such that the total length of all contigs not shorter than this is at least half the total length of all contigs in the assembly; L50^*^ - minimum number of contigs whose total length is at least half the total length of the assembly; HTG - predicted potential events of possible horizontal gene transfer.

### 3.3 Phylogeny and comparative analysis of annotated genomes

**Figure 4:**
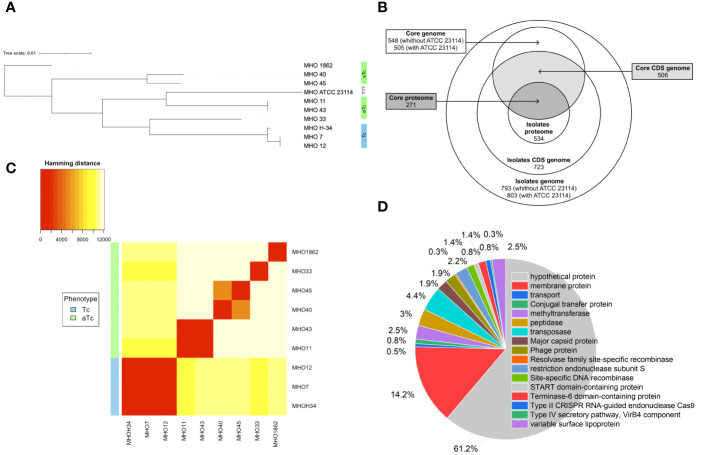
**(A)** phylogenetic tree between clinical isolates and the laboratory strain MHOH34 of *M. hominis* based on genome-wide sequencing and comparative genome analysis, **(B)** Venn diagram showing the total number of genes and proteins (pangenome and panproteome), as well as the core genome and proteome, **(C)** clustering of clinical isolates by SNP in genomes, **(D)** functional analysis of unique genes found only in clinical isolates and therefore missing from the genome of the laboratory strain MHOH34.

For constructing a phylogenetic tree, MHOH34 and ATCC23114 (PG21) was used as the reference strain. Isolates MHO12 and MHO7 are closely related to the laboratory strain MHOH34, and isolate MHO11 – to the reference strain ATCC23114. Isolates MHO11 and MHO43, as well as MHO40 and MHO45, are closely related to each other, while MHO1862 and MHO33 differ from all others (**Figure 4A**). Clustering of all studied strains by SNP showed that all isolates are distributed into groups, with isolates forming TCs (MHOH34, MHO12, MHO7) clustering into one group, and all other clinical isolates, characterized by the formation of aTCs, are grouped into separate groups with pairwise similarity for MHO11 and MHO43 and for MHO40 and MHO45, with isolates MHO1862 and MHO33 being positioned separately. Genome alignments were conducted using the MAUVE program (**Supplementary Figure S1**). The size of the genomes of the strains corresponded to the size of the genome of other fully sequenced isolates of *M. hominis* available in databases and ranged from 681,052 to 757,124 bp. The alignment process identified 11 locally collinear blocks, representing homologous DNA segments common to the strains without sequence rearrangements. Genome annotation was performed using Bakta and Blast programs. Despite the small number of studied isolates, we were able to determine the core genome and pangenome (**Figure 4B**; **Supplementary Figure S2**). The pangenome for the group of studied isolates comprises 803 genes. The core genome consists of 505 genes. The number of common and unique genes for each strain is shown on the Upset Plot (**Supplementary Figure S3**). Functional analysis of genes unique to clinical isolates (those who are absent in the laboratory strain) is presented in **Figure 4D**. Among them, the highest percentage are genes coding for proteins of unknown function (61.2%) and lipoproteins (14.2%), particularly variable membrane proteins. We also identified genes unique to each of the strains. For instance, the laboratory strain MHOH34 has one unique protein-coding gene MHOH34_02430, annotated as a lipoprotein. Isolates MHO45, MHO40, and MHO12 have three unique genes, annotated as lipoproteins, hydrolase TatD (MHO40), and genes coding for proteins of unknown function. Isolate MHO43 has two unique genes coding for proteins of unknown function. The largest number of unique genes is found in the genomes of isolates MHO1862 and MHO33. Most of them are genes coding for proteins of unknown function. In addition, in the case of MHO1862, there are genes annotated as conjugal transfer protein, VirB4 component type IV secretory pathway, cell wall-associated hydrolase, NlpC_P60 family, conjugative transposon protein TcpC, tetracycline resistance ribosomal protection protein Tet(M), XRE-family HTH domain contain transcriptional regulator, transposase, ABC-type multidrug transporter, Na+-driven multidrug efflux pump norM, dinF/norM/MATE family protein, Sigma70-r4 domain-containing protein, three recombinases, Dam family site-specific DNA-(adenine-N6) methyltransferase, and three lipoproteins. In the case of MHO33, there are 28 unique genes. Besides genes coding for proteins of unknown function, the genome composition includes Site-specific DNA-methyltransferase (adenine-specific), Arc-trans-TRASH domain-containing protein, Sugar-phosphate nucleotidyltransferase, Conjugal transfer protein, Single-stranded DNA-binding protein, and DUF1508 domain-containing lipoprotein. We also discovered common unique gene patterns. Isolates MHOH34, MHO12, and MHO7 have the same unique set of genes, including ribonuclease, Lmp protein, three methyltransferases, three 5-methylcytosine-specific restriction endonucleases, two variable surface lipoproteins, two AAA family ATPases, peptidase-C39-2 domain-containing protein, XRE family transcriptional regulator, phg-2220-C domain-containing protein, phage-int-SAM-5 domain-containing protein, protease Clp, and transposase. We also found the same pattern of genes unique to isolates MHO11 and MHO43. Besides 24 genes coding for proteins of unknown function, this includes two genes coding for type I restriction endonuclease subunits, as well as site-specific DNA-methyltransferase, HTH luxR-type domain-containing protein, LPD29 domain-containing protein, transposase, and two lipoproteins.

Pearson clustering based on genome annotation allows for the distribution of all strains into the same groups as the ones identified based on SNP clustering. The genomes of isolates MHO1862 and MHO33, which have the largest number of unique genes in their genome composition, cluster separately from all other strains (**Figure 4C**). Since clinical isolates were more resistant to ofloxacin compared to the laboratory strain (except for MHO12), we compared the amino acid sequences of gyrase GyrA, GyrB, and topoisomerases ParE and ParC relative to the laboratory strain (**Supplementary Table S1**). Isolates MHO11, MHO43, MHO33 and MHO1862 showed significant resistance to the ofloxacin. This may be related to the amino acid substitutions in topoisomerases ParE (V154A in MHO33, N209D in MHO1862), ParC (N542S, M623I, N828D, E862Q in MHO33, R144K, M623I, A738T, D861N in MHO1862 and F484L, N542S, N898D in MHO11 and MHO43), GyrA (R79K, E272K in MHO11 and MHO43) and GyrB (S302A, V385I in MHO11 and MHO43) compared to the laboratory strain.

### 3.4 Analysis of mobile elements

The analysis of the presence of mobile genomic elements (MGEs)was conducted using Proksee software (**Supplementary Figure S4**, **Supplementary Table S2**). Unlike the laboratory strain, many horizontal gene transfer (HTG) events were predicted for all isolates. Isolate MHO1862 stands out in terms of the number of unique MGEs. It alone contains the conjugative transposon ICE (integrative and conjugative element), which has some similarity to ICEB1 found in Bacillus subtilis. It includes a system of conjugative elements, the antirestriction protein ardA, serine recombinase pinR, excisionase Xis, integrator complex subunit int, and transcriptional regulator immR_1. The regulation of ICEBs1 is carried out by integrase Int, transcription repressor ImmR, and metallopeptidase antirepressor ImmA (Lee et al., 2007). ImmR acts as a repressor that ensures a single stable copy of ICEBs1 is maintained in the cell in a dormant state. If a global DNA damage response is triggered or if nearby cells—potential recipients lacking the transposon—are present, ImmA inactivates ImmR through proteolytic cleavage, which initiates the expression of ICEBs1 and facilitates the transfer of the transposon. Such integrative and conjugative elements can spread among bacteria via conjugation, thus contributing to genome plasticity and the dissemination of antibiotic resistance and virulence factors among species. Isolates MHO43 and MHO11 contain additional genes of the RM system type I, *hsdM*; MHO43 also has an additional gene, *hsdS*. Through genome annotation, we were able to expand the number of identified MGEs in the genomes of clinical isolates. It was found that isolate MHO1862 contains the gene *tcpC* of the *tcp* transfer locus from the known conjugative plasmid pCW3, which codes for tetracycline resistance. TcpC protein is necessary for effective conjugative transfer and is localized on the cell membrane independently of other conjugating proteins. It has been shown to form oligomeric complexes facilitating efficient gene transfer. Its homology with the type IV secretion system component VirB8 of *Agrobacterium tumefaciens* was determined (Porter et al., 2012). MHO1862 also contains genes of the type IV secretion system component *virB4*, tetracycline resistance ribosomal protection protein Tet(M), and START domain-containing protein. The latter belongs to a family of proteins involved in lipid transport. Isolates MHO33, MHO40, MHO43, MHO11, and MHO1862 contain phage genes (genes coding terminase-6 domain-containing protein, *Mycoplasma* phage MAV1, Phg-2220-C domain-containing protein, Phage-int-SAM-5 domain-containing protein) and viral gene (coded major capsid protein), as well as Type II CRISPR RNA-guided endonuclease Cas9 and DJ-1/PfpI family protein, which is analogous to YajL of *Escherichia coli* and known as an oxidative stress sensor. Phage MAV1 was previously found in the genome of *Mycoplasma arthritidis* (Voelker and Dybvig, 1998). Isolates MHO12, MHO7, MHO33, MHO45, MHO11, and MHO1862 contain genes coding for Holliday junction proteins (Holliday junction resolvases RecU, RuvX, Holliday junction branch migration protein RuvA, branch migration DNA helicase RuvB), involved in the formation of Holliday junction DNA during genetic recombination and DNA repair. The absence of RuvAB, RecU or RuvX in some of the clinical isolates likely due to genome incompleteness or mis annotations. These same isolates contain a gene coding for ComEC/Rec2 family competence protein, involved in the binding and uptake of DNA during transformation.

### 3.5 Identification of restriction-modification system genes

**Figure 5:**
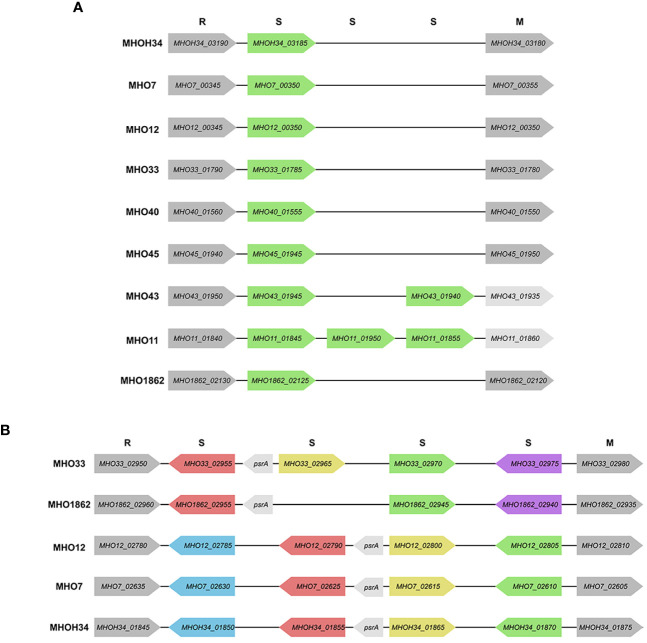
Scheme of *hsd1* locus **(A)** and *hsd2* locus **(B)**, featuring the position and direction of Type I restriction modification system genes (R – endonuclease *hsdR*, S – DNA-specific subunit *hsdS*, M – methyltransferase *hsdM*), *psrA* -tyrosine-type DNA invertase. The same color indicates the homology of the genes.

Since additional genes coding for subunits of restriction-modification (RM) systems were found in clinical isolates, we decided to identify all genes related to RM systems and compare how the isolates differ in terms of the number and composition of RM genes. Two type I RM system loci, designated as hsd1 and hsd2, were identified in the genomes of isolates and the laboratory strain MHOH34. All studied strains have the hsd1 system, which includes an endonuclease subunit (HsdR), a methyltransferase subunit (HsdM), and a DNA-specific subunit (HsdS) with homologous sequences, except for MHO11 and MHO43 (they have additional HsdS (**Figure 5A**). Only 5 of the studied strains have the second hsd2 system, located in another part of the genome - MHOH34, MHO7, MHO12, MHO33, and MHO1862 (**Figure 5B**). Three of them (MHOH34, MHO7, MHO12) are completely homologous, each having one hsdM, hsdR, and 4 hsdS, which form inverted repeats in the locus. The hsdR, hsdM, and one of the hsdS genes are coded in tandem on the same DNA strand. Isolate MHO33 has 4 hsdS, MHO1862 – 3 hsdS, each of them non-homologous to the S-subunits in isolates MHOH34, MHO7, MHO12. All isolates have genes psrA, coding for tyrosine-type DNA invertase, between S-subunits, which can cause inversion, generating multiple recombinant S-subunit genes with alternative DNA-binding specificity (Li et al., 2020).

The functional role of type I RM systems for *M. hominis* is not yet studied. However, for *Mycoplasma pulmonis*, it was shown that phase variations occur at the level of the *hsdS* gene through reversible recombination processes acting on multiple *hsdS* alleles, allowing for changes in methylation target specificity and therefore switching the bacteria between alternative DNA methylation patterns. It is suggested that the potential for phase-variable DNA methylation allows the bacterium to alter its phenotype in response to external environmental shocks, serving as a regulatory mechanism in bacteria (Gumulak-Smith et al., 2001). In addition to these two systems, additional *hsdM*, *hsdR*, and *hsdS* were found in the genome of clinical isolates (**Supplementary Table S2**).

Based on the presence of homologous RM system subunits, our isolates can be divided into groups. The first group, separate from the others, consists of MHOH34, MHO12, and MHO7; they have the same set of RM system subunits, and all form TCs on agar. The rest of the isolates form the second group, all forming aTCs on agar. This group can also be distributed: nearly identical sets of RM subunits are found in MHO43 and MHO11, MHO33 and MHO1862, as well as MHO40 and MHO45.

### 3.6 Comparative proteomic analysis of the laboratory strain MHOH34 and eight clinical isolates of *M. hominis*

After comparing the genomes, we conducted a comparative proteomic analysis of the clinical isolates and the laboratory strain using LC-MS. Protein identification was carried out relative to the annotated and assembled genome of the laboratory strain MHOH34. The number of identified proteins is shown in **Table 3**. The total number of identified proteins in all strains is 518, with 271 proteins common among all studied strains (**Figure 4B**; **Supplementary Figure S5**).

**Table 3:** Number of identified proteins in clinical isolates of *M. hominis*, as well as the number of differentially expressed proteins in clinical isolates compared with the laboratory strain MHOH34.

*M. hominis* strain	number of identified proteins	number of identified proteins with cut-off pvalue ≤ 0.05	number of differentially expressed proteins with cut-off p value ≤ 0.05 and FC= ± 1
MHO7	390	305	138
MHO45	372	188	117
MHO40	387	255	139
MHO12	384	242	67
MHO1862	381	284	116
MHO11	387	268	124
MHO43	387	198	107
MHO33	368	199	109

**Figure 6:**
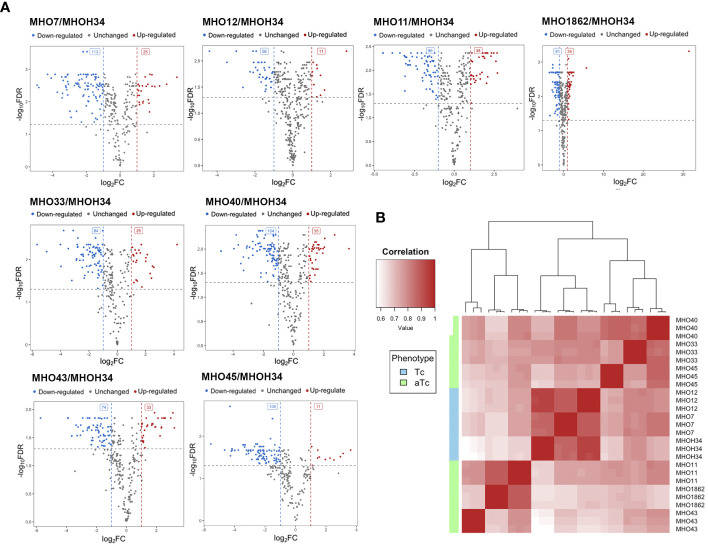
**(A)** Pearson clustering in accordance with the protein abundance in the laboratory strain MHOH34 and in clinical isolates of *M. hominis* (MHO12, MHO7, MHO33, MHO40, MHO43, MHO45, MHO11, MHO1862). **(B)** volcano plots indicating the number of differentially expressed proteins in eight clinical isolates compared with the laboratory strain MHOH34.

A comparative quantitative analysis of the proteomes of the eight clinical isolates and the proteome of MHOH34 was conducted using the Quantms next flow pipeline software package (**Supplementary Table S4**), which showed that the clinical isolates significantly differ in protein abundance and composition compared to the laboratory strain (**Figure 6A**). Based on the obtained proteomic data, Pearson clustering allowed for the stratification of strains into groups. As in the case of comparative genome analysis, the laboratory strain MHOH34 and clinical isolates MHO12 and MHO7 cluster into a separate group from all others (**Figure 6B**). Functional analysis of differentially expressed proteins, reliably identified (data presented with a cutoff by pvalue=0.05 and FClog2 = 1) for each clinical isolate (**Supplementary Figure S6**), identified patterns of proteins whose representation changes in all clinical isolates. Among the proteins whose abundance decreases by 2 or more times compared to the laboratory strain there are proteins associated with cell division (FtsZ, MraZ, ObgG (**Figure 2D**), ATP synthase subunits, proteins involved in replication and translation processes, membrane and transport proteins, as well as enzymes of energy metabolism. The decline in the level of proteins associated with major energy-intensive cellular processes aligns with the reduced growth rate of clinical isolates compared to the laboratory strain, which may be a common step toward energy conservation. Alongside, the increase in abundance of ribosomal proteins - components of both large and small ribosomal subunits, featured in all isolates (**Supplementary Figure S6**), may result from incomplete synchronization between cell division and other cellular processes. However, there is increasing evidence that ribosomal proteins, besides their canonical functions, may serve other roles, such as acting as nucleoid-binding proteins (Fisunov et al., 2021) or functioning as ligands for ribosome switching (Deiorio-Haggar et al., 2013). It cannot be excluded that the upregulation of ribosomal proteins in all isolates is somehow related to the adaptation of *M. hominis* in the host organism. Besides ribosomal proteins, we observe an increase in abundance of some membrane proteins, variable antigens or immunoglobulin-binding lipoproteins, enzymes of nucleoside metabolism, oxidoreductases, proteases, proteins involved in RNA processing and ribosome biogenesis (**Supplementary Figure S6**). It is noteworthy that almost all isolates show an increased abundance of the DegV protein, which binds fatty acids with high affinity. *Mycoplasmas* have limited metabolic capabilities and strictly depend on the host organism or nutrient medium, particularly in terms of the supply of exogenous fatty acids, as they cannot synthesize them. Fatty acids are necessary for the topology, fluidity, permeability, and integrity of the cell membrane, which, in turn, has significant implications for the adaptation of *Mycoplasma* to external conditions. DegV may also participate in the transport of fatty acids. Proteins whose abundance increases exclusively for one of the isolates were also identified. For isolate MHO33, the abundance of the MetK protein, responsible for the biosynthesis of S-adenosylmethionine synthase, a cofactor involved in methyl group transfer, increases. For isolates MHO43 and MHO45, there is an increase in the level of phosphatase Ppa, and for isolate MHO1862, a significant (FC=31.46) increase in HAD-IIB family hydrolase of unknown function.

**Figure 7:**
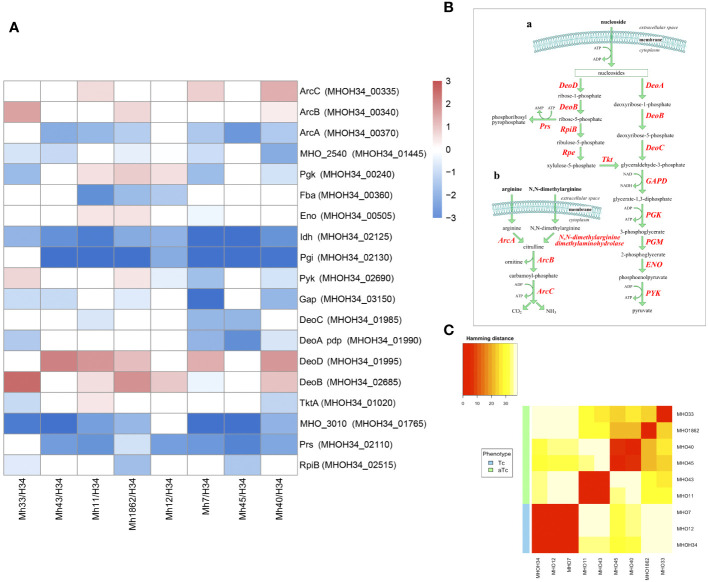
**(A)** differentially expressed proteins of the energy metabolism of clinical isolates MHO12, MHO7, MHO33, MHO40, MHO43, MHO45, MHO11, MHO1862 of *M. hominis* compared with the laboratory strain MHO34; **(B)** the scheme of energy metabolism of *M. hominis*, **(C)** Pearson clustering based on abundance protein in the laboratory strain MHO34 and clinical isolates of *M. hominis*. White boxes are the proteins with non-significant changes.

Previous studies have shown that the phenotype of *M. hominis* colonies is related to the switch in energy metabolism: for *M. hominis* cells forming TCs and characterized by high growth rate, the use of the most energetically favorable pathway associated with arginine utilization is typical, while for *M. hominis* cells forming slowly growing aTCs, there is a switch to a less energetically favorable pathway associated with the utilization of nucleosides as a carbon source (Fisunov et al., 2022). In this study, we were interested in tracing which energy metabolism pathway is most active in clinical isolates based on the obtained proteomic data. It is known that several pathways of energy metabolism operate in *M. hominis*: trunk pathway of glycolysis (Desantis and Pollack, 1989), part of the pentose phosphate pathway (Arraes et al., 2007), the arginine dehydrogenase pathway (Pereyre et al., 2009), and the recently discovered nucleoside utilization pathway (Fisunov et al., 2022). Since *M. hominis* lacks the enzymes glucokinase and 6-phosphofructokinase, *M. hominis* cells cannot efficiently use glucose as a carbon source (Halbedel et al., 2007). Additionally, it has an incomplete set of proteins for the phosphoenolpyruvate-dependent phosphotransferase system (PEP-PTS), with only two components annotated - putative energy-coupling protein HPr and component B of the enzyme II complex (Glass et al., 2000). Instead of glucose, it uses glyceraldehyde-3-phosphate, which is formed as a result of the pentose phosphate pathway. The arginine dehydrogenase pathway is used by *M. hominis* as an alternative energy source, necessary for initiating growth (**Figure 7B** (b)). Arginine is utilized using 4 enzymes – arginine deiminase (ArcA), N(G),N(G)-dimethylarginine dimethylaminohydrolase, ornithine carbamoyltransferase (ArcB), and carbamate kinase (ArcC). In the process of nucleoside utilization, 4 enzymes participate: two of them - nucleoside phosphorylases DeoD and DeoA - catalyze the cleavage of ribonucleosides and deoxyribonucleosides into bases and ribose-1-phosphate or deoxyribose-1-phosphate, respectively (**Figure 7B** (a)). The bases are subsequently used for nucleotide synthesis or serve as a nitrogen source. Ribose-1-phosphate can be converted into ribose-5-phosphate by the enzyme phosphopentomutase DeoB and then into phosphoribosyl pyrophosphate, which is used for *de novo* nucleotide synthesis or further utilized in the pentose phosphate pathway. Deoxyribose-1-phosphate, formed from deoxyribonucleosides, can also be converted by DeoB into deoxyribose-5-phosphate, which is degraded by the enzyme deoxyriboaldolase DeoC into glyceraldehyde-3-phosphate, which, in turn, can be further utilized in the glycolysis process. Comparative analysis of the proteomes showed that despite all strains being grown with arginine added to the culture medium as a carbon source, all clinical isolates, unlike the laboratory strain MHOH34, tend to have a decreased level of glycolysis enzymes (we observe downregulation of Gap and Pgi and in some isolates – Pgk and Pyk), arginine dehydrogenase pathway (the abundance of enzymes of the first limiting reaction of arginine conversion - ArcA and N(G),N(G)-dimethylarginine dimethylaminohydrolase decreases) and the pentose phosphate pathway (downregulation of RpiA, TktA, and phosphoketolase MHOH34_01765) (**Figure 7A**). Downregulation of ribose-phosphate pyrophosphokinase PrsC, which directs the metabolic flow towards *de novo* nucleotide synthesis by converting ribose 5-phosphate into phosphoribosyl pyrophosphate (PRPP), indicates that the activity of this pathway is also reduced. Among the enzymes of nucleoside utilization, we observe a decrease in the abundance of DeoA and DeoC, which are involved in the utilization of deoxyribonucleosides. However, an increase in the abundance of DeoD and DeoB indicates that for clinical isolates, the activation of the energy flow towards ribonucleoside utilization, which among all energy-gaining pathways available to *Mycoplasma*, is the least favorable [**Figure 7B** (a)]. It can be assumed that the reduced growth rate of clinical isolates, accompanied by a reduction in colony size, is related to their selection of the least energetically favorable carbon metabolism pathway associated with the utilization of ribonucleosides. This allows them to slow down growth and transition to a state similar to persisters, which may contribute to survival under stress conditions. Even passaging isolate through 2 passages on a rich medium with arginine does not completely switch the metabolism to a more energetically favorable arginine dehydrogenase pathway. The activity of energy metabolism enzymes may depend on amino acid substitutions in the active sites of enzymes. We found that clinical isolates forming aTCs have amino acid substitutions in enzymes of the arginine dehydrogenase pathway (ArcA, ArcB, ArcC), glycolysis (Gapd, Eno, Pgk, Pgm, Pyk), and the nucleoside utilization pathway (DeoA, DeoD) compared to the laboratory strain and isolates forming TCs (**Supplementary Table S5**). Pearson clustering based on amino acid substitutions allows for the stratification of isolates into groups: isolates forming TCs (MHOH34, MHO12, MHO7) cluster into one group, while all other clinical isolates, characterized by the formation of aTCs, are grouped into separate groups with pairwise similarity for MHO11 MHO43 and for MHO40 and MHO45, isolates MHO1862 and MHO33 are positioned separately (**Figure 7C**). We noted that the substitution F188Y in the DeoD protein is present in clinical isolates forming aTCs, and this significant substitution is located close to the enzyme’s active site and may affect its activity (Pugmire and Ealick, 2002).

**Figure 8:**
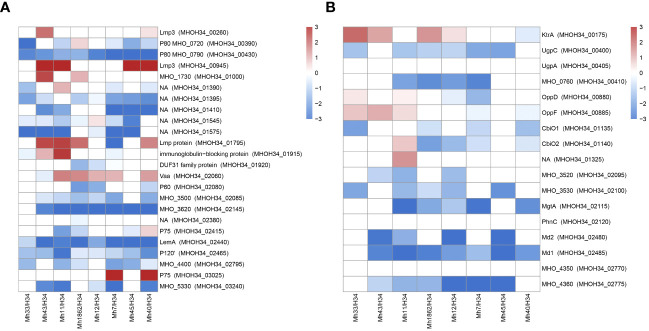
Differentially expressed membrane proteins **(A)** and transport proteins **(B)** of *M. hominis* clinical isolates MHO12, MHO7, MHO33, MHO40, MHO43, MHO45, MHO11, MHO1862 of *M. hominis* compared to the laboratory strain MHO34. White boxes are the proteins with non-significant changes.

Another functional group of interest for study are membrane proteins. *M. hominis* lacks a cell wall, so the membrane is the primary mediator facilitating its interaction with the environment. *Mycoplasma* lipoproteins perform a multitude of functions, ranging from nutrient transport or substrate hydrolysis to adhesion, virulence, and immunomodulatory activity (Christodoulides et al., 2018). Proteomic analysis showed that clinical isolates significantly restructure the abundance of membrane proteins compared to the laboratory strain (**Figure 8A**). We observe some general patterns of changes in membrane proteins for isolates: an increase in the variable antigen Vaa is characteristic of all isolates (except MHO43, where it remains unchanged). Vaa was reliably identified in the proteome of isolates MHO7, MHO11, MHO12, MHO40 and MHO1862, and for isolates MHO33 and MHO45 – among the unique proteins. For isolates MHO43, MHO11, MHO45, and MHO40, there is a significant increase in the abundance of Lmp3 (MHOH34_00945). Meanwhile, all isolates show a decrease in the abundance of lipoprotein P80 (MHOH34_00430). For most isolates, there is an increase in the level of Lmp protein (MHOH34_01795) and a decrease in the level of LemA (MHOH34_02440), variable lipoproteins MHOH34_02795, P120’, LemA, and MHOH34_01395. In MHO40 and MHO7, the level of P75 (MHOH34_03025) increases and two isolates MHO11 MHO43 show an increase in the level of immunoglobulin-binding protein MHOH34_01915. Clinical isolates were obtained from patients with urogenital infections therefore we assume that the downregulation of some lipoproteins and upregulation of others is related to adaptation in the host organism. The level of another lipoprotein MHO_3200, which contains a peptidase domain DUF31, belonging to the endopeptidase superfamily and presumably playing a role in modulating the action of the host’s innate immunity, decreases in MHO1862. Vaa is a variable antigen and also participates in adhesion to host cells, its upregulation is characteristic for all clinical isolates, except MHO43. It is known that the diversity of genome-encoded Vaa surface lipoproteins depends on: the loss of multiple repeats (about 363 bp), located in the central part of the gene coding for the alpha-helical region, frameshift mutations, and due to deletions or nucleotide substitutions in the variable C-terminal region of the gene (Zhang and Wise, 1996). We have previously compared the sequences of the *vaa* genes of the laboratory strain MHOH34 and 7 clinical isolates of *M. hominis* (Galyamina et al., 2024), showing that unlike the laboratory strain and isolates MHO12 and MHO7 (forming TCs), clinical isolates forming aTCs have significant amino acid substitutions in Vaa. Mostly, they are located in the variable C-terminal part of the protein, which may affect the ability to attach to eukaryotic cells and by that evade the immune response. In this study, we added another isolate MHO33 and compared their sequences again (**Supplementary Figure S7**). The diversity of *vaa* is determined by the variable number and composition of homologous interchangeable cassettes, located in the C-terminal part of the protein and approximately 110 amino acid residues in length (Boesen et al., 2001). The cassette organization of *vaa* arises as a result of combinations of cassette duplications and deletions in some *M. hominis* isolates and cassette recombination between isolates. Besides MHO33, all studied strains have a Vaa sequence length of 344-348 amino acids and have 2 interchangeable cassettes. Isolate MHO33 has three interchangeable cassettes and a longer sequence (466 amino acids). In the C-terminal variable region, it also has a significant number of meaningful amino acid substitutions. Based on amino acid substitutions, all isolates can be clustered into three groups: one group consists of isolates MHO11 and MHO43, having the same nucleotide substitutions in the C-terminal region of the gene and a shorter gene size compared to all isolates except MHO33. The other two groups consist of isolates MHO45, MHO40, MHO1862 and isolates MHOH34, MHO12, and MHO7 (Galyamina et al., 2024). Interestingly, this distribution into groups coincides with the clustering of strains by colony phenotype. Variability in molecular weight and amino acid sequences of Vaa may affect adhesion and the ability to evade the immune response (Saadat et al., 2018).

Transport proteins represent another functional group of interest. We observe a significant decrease in the level of transport proteins in all clinical isolates. This included ABC transporters, which are required for transport of a number of different substrates. The transport system in *Mycoplasmas* is ATP-dependent and a reduction in energy metabolism activity undoubtedly affects its functioning. Against the backdrop of downregulation, we observe upregulation of certain transporters. In isolates MHO43, MHO11, MHO45, and MHO40 among unique proteins, we identified the transporter BcrA, which presumably has a binding site for nucleosides and could be a candidate for nucleoside transport. For isolates MHO33, MHO43, MHO12, and MHO1862, there is an upregulation of KtrA, which is responsible for transport of potassium ions. All isolates, except MHO33 and MHO12, show an increase in the abundance of transport protein MHOH34_03095, and for isolates MHO11, MHO7, and MHO40 – MHOH34_3100. Isolates MHO33, MHO43, and MHO11 show an increased abundance of Peptide ABC transporter OppD and OppF (**Figure 8B**).

We found that among the unique proteins (identified in all clinical isolates but not detected in the proteome of the laboratory strain), one component of the type I restriction-modification (RM) system – the methyltransferase HsdM (MHOH34_03180) is present in all clinical isolates (**Supplementary Table S4**). Meanwhile, the abundance of the nuclease subunit HsdR and the recognition subunit HsdS decreases or remains unchanged. In the case of isolate MHO1862, there is an increase in the abundance of Type III restriction-modification system methylase Mod, which was annotated in the genome of this isolate and detected in the proteome. It is known that RM systems primarily function to protect against the intrusion of foreign DNA, however, it has also been shown that they can perform non-classical functions such as participating in phase variations and gene expression regulation (Ershova et al., 2015; Sitaraman, 2016; DebRoy, 2021). It is suggested that the methyltransferase component of RM systems may regulate gene expression, particularly the expression of virulence factor genes in bacterial pathogens.

**Figure 9:**
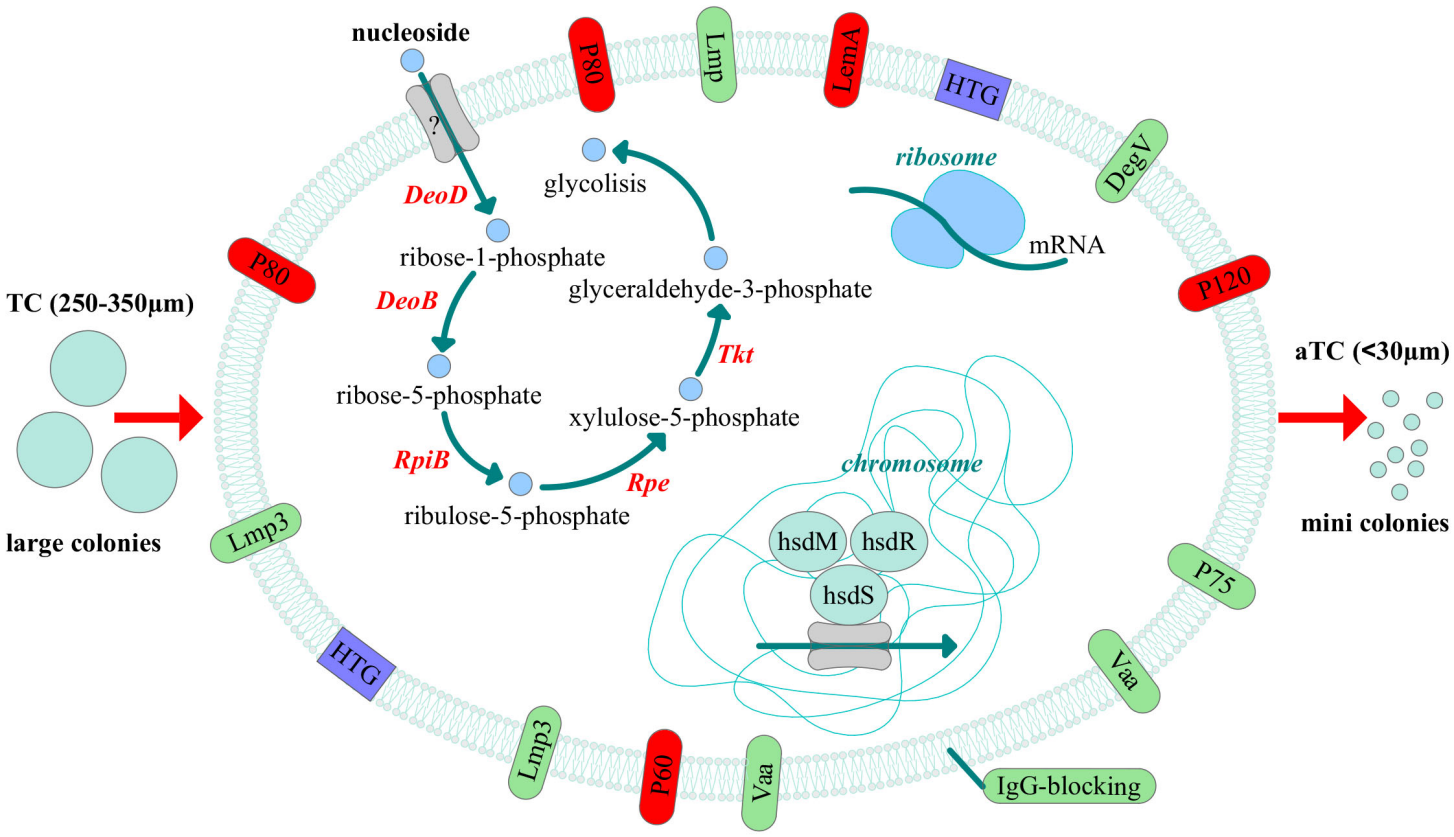
Adaptive strategies of clinical isolates of *M. hominis* revealed by comparative proteogenomic analysis with a laboratory strain MHOH34. The pathway of nucleoside utilization, changes in the abundance of membrane proteins, horizontal gene transfer (HTG) events and the possible participation of the type I restriction modification system and ribosome proteins in the regulation of adaptation in the host are shown. All these events can lead to the phenotypic difference, namely reduced size of the colonies. The down-regulated proteins are indicated in red and up-regulated - in green.

Thus, we discovered that clinical isolates differ from the laboratory strain in their phenotypic characteristics. They form colonies of different sizes on solid agar, primarily atypical mini colonies (aTCs) for *Mycoplasmas*, except for two isolates, MHO12 and MHO7. Phenotypic differences in *M. hominis* colonies when seeding biological material from patients with various pathologies have been observed before (Bragina et al., 2023). The difference in colony phenotype of clinical isolates may be related to their isolation from patients with different medical histories, treatment methods, and degrees of infection chronicity. Apart from MHO12, all clinical isolates grow significantly slower in a liquid medium than the laboratory strain, more effectively form biofilms, and exhibit resistance to ofloxacin. We believe all these phenotypic features are related to adaptation within the host organism. Proteogenomic profiling and comparative analysis of clinical isolates with MHOH34 identified several strategies *Mycoplasmas* use to survive in competitive and adverse environments (**Figure 9**). Despite all strains being grown in a rich medium with arginine as a carbon source, most isolates showed reduced growth rate and smaller colony sizes, which, according to proteomic analysis data, was accompanied by downregulation of proteins involved in cell division, replication, translation, and arginine utilization enzymes. This suggests that the change in the phenotype of clinical isolates is related to a functional restructuring towards a starvation-like state, akin to persisters. Based on proteomic data, we hypothesize that the most energetically unfavorable carbon metabolism pathway, the ribonucleoside utilization pathway, is activated in clinical *M. hominis* isolates, allowing them to slow down growth and maintain only basic cellular processes. This phenomenon of metabolic switching was previously observed in our study on the formation of *M. hominis* mini colonies, characterized by slow growth and antibiotic resistance (Fisunov et al., 2022). In the host organism, such a phenotypic switch aids survival in competitive and adverse environments. A similar persister-like phenotype of *Mycoplasmas* was first discovered in the works of I.V. Rakovskoy et al. *Mycoplasmas* have been found to form mini-colonies when exposed to stress. Very slow growth and resistance to adverse factors were their main properties (Rakovskaya et al., 2019).We identified a large number of amino acid substitutions in the sequence of energy metabolism enzymes in clinical isolates, which could affect the efficiency of enzyme activity. Specifically, we noted that all clinical isolates forming aTCs have an F188Y substitution in the DeoD protein, located close to the enzyme’s active site involved in ribonucleoside utilization. It cannot be excluded that this substitution may play a role in enzyme activity and contribute to the activation of the ribonucleoside utilization pathway, characteristic of aTCs-forming clinical isolates. Clinical isolates form biofilms more effectively compared to the laboratory strain, which is also associated with slowed growth and resistance to adverse environments. The ability of *Mycoplasmas* to colonize the respiratory and urogenital tracts of humans to form biofilms has been shown before (Feng et al., 2021). The formation of a persisting phenotype is also supported by the known difficulties in cultivating clinical *M. hominis* isolates, which grow poorly even on the nutritious media. Perhaps, clinical isolates are so adapted to life within the host organism that they cannot easily be switched to a state of actively replicating cells. The reduction in metabolic activity is a common strategy for bacteria to survive in the host organism (Koczula et al., 2017; Gray et al., 2019), and for *Mycoplasmas* with a reduced genome, it may be one of the main adaptation mechanisms.

Along with slowed growth, the repertoire of membrane proteins in clinical isolates changes compared to the laboratory strain, which may also be an additional strategy for adaptation in unfavorable conditions. *Mycoplasmas* have a high proportion of genes coding for lipoproteins (about 10%), many of which are involved in interaction with host cells and protection against the immune response (Christodoulides et al., 2018). It has been shown that the expression of genes coding for membrane proteins significantly changes during interaction with eukaryotic cells, as well as under osmotic and oxidative stress. The exact type of membrane proteins, detected in the proteome, also varies in different strains. (Hallamaa et al., 2008; Matyushkina et al., 2016). The variable structure of surface membrane proteins P120, Lmp, and Vaa helps evade the host’s immune system action (Ladefoged et al., 1996; Ladefoged and Christiansen, 1998; Zhang and Wise, 2001). The *vaa* gene product participates in adhesion to host cells (Zhang and Wise, 1996). The diversity of antigens is achieved through various means: variability of the *p120* gene is achieved through the accumulation of mutations in a specific gene region, for *lmp* it is characterized by gene size changes, and for the *vaa* gene, both size changes and frame-shifting mutations to create various products (Boesen et al., 1998). Numerous specific proteins such as P50, P60, P80, and P100 on the surface of *M. hominis* play an important role in the attachment of bacteria to host cells, which is necessary for their colonization and survival (Henrich et al., 1993; Kitzerow et al., 1999). In our study, clinical isolates exhibit upregulation of Vaa, Lmp protein (MHOH34_01795), and for four of them (MHO43, MHO11, MHO45, and MHO40) – upregulation of Lmp3. The high abundance of these proteins in clinical isolates may reflect their significant role in adaptation to life inside the host organism. Clinical isolates (except of MHO12 and MHO7) differ from the laboratory strain in size and a larger number of nucleotide substitutions in the variable C-terminal sequence of the *vaa* gene, which may affect adhesion abilities and evasion from the immune response (**Supplementary Figure S7**).

The upregulation of ribosomal proteins alongside with the downregulation of all other cellular processes in clinical isolates is intriguing. It may be related to a disruption in synchronization between cell division and main cellular processes. We found that the abundance of the transcription factor MraZ, which regulates the link between cell division and metabolism (Fisunov et al., 2016), is reduced in some clinical isolates compared to the laboratory strain. The phenomenon of ribosomal protein upregulation was observed by Troy Sandberg et al., who studied how the genomic and transcriptomic profile changes over many generations in the JCVI-syn3.0 strain, the first living organism with a minimal synthetic genome based on *Mycoplasma mycoides* genome (Sandberg et al., 2023). The strain was characterized by very slow growth. Similar to our case, the authors observed enhanced regulation of ribosomal proteins and reduced regulation of DNA and RNA-related proteins during adaptation. It is not clear what role ribosomal proteins may play in the adaptation of clinical isolates in the host organism and how this may be related to growth rate. Ribosomal proteins are known to perform non-canonical functions. Fisunov et al. suggested that ribosomal proteins non-specifically bind DNA and participate as nucleoid-associated proteins, regulating nucleoid architecture, which in turn affects gene expression regulation (Fisunov et al., 2021). Since *Mycoplasmas* have a minimal number of known transcription factors, gene regulation may be carried out by other mechanisms, in particular by changing the structure of nucleoid. Another study showed that some ribosomal proteins can bind simple RNA structures and serve as ligands for ribosome switching (Deiorio-Haggar et al., 2013). It has also been shown that ribosomal proteins can play a role in antibiotic resistance. Point mutations in the genes of some ribosomal proteins are responsible for acquiring resistance to macrolides and lincosamides (Prats-van der Ham et al., 2018; Na Wang et al., 2023). It is possible that in the case of clinical isolates, the upregulation of ribosomal proteins plays an important role in their adaptation to host and requires further study of its role in adaptation.

It was noteworthy, that methylating subunits HsdM of the type I RM system was upregulated in all clinical isolates. We also identified additional components of RM systems in isolates. Such diversity of RM system components in an organism with a very small genome size suggests their possible involvement in regulating adaptation processes. The role of DNA methylation in gene regulation has long been known, but for *Mycoplasmas*, it was first demonstrated only in 2013 (Lluch-Senar et al., 2013). Analysis of the distribution of methylated motifs and their functional significance in strains of *M. genitalium* and *M. pneumoniae* showed a potential regulatory role of methylation in the cell cycle and gene expression. The same role of DNA methylation was shown for *M. agalactiae* (Dordet-Frisoni et al., 2022). Experimentally, it has been shown that some RM systems play an important role in the conjugative horizontal DNA transfer of *Mycoplasma*, i.e., DNA methylation can not only contribute to the regulation of vital biological functions at the cellular and population levels but also plays a significant role in the evolution and adaptation of the *Mycoplasma* genome, controlling horizontal gene transfer (HGT).

It cannot be excluded that some changes in the proteome of different isolates may be associated with differences in the growth phase from which they were collected.

Genomic analysis showed that clinical isolates of *M. hominis* possess a fairly large diversity of genes within a single species; the pangenome for the 9 studied strains consists of 723 protein-coding genes, and the core genome – 506. The percentage of genes unique to the isolates amounts to 30%. Unlike the laboratory strain, clinical isolates possess a set of MGEs such as: phage genes, viruses, Type II CRISPR RNA-guided endonuclease Cas9, numerous recombinases and integrases, as well as integrative conjugative elements (ICEs) (**Supplementary Table S2**). ICEs are self-transmissible mobile genetic elements and key mediators of HGT. For a long time, it was believed that the only driving force of *Mycoplasma* evolution was the successive loss of genetic material due to a parasitic lifestyle, and only recently has it been proven that HGT is possible in *Mycoplasmas* (Dordet-Frisoni et al., 2014). Recently, ICEs have been found in the genomes of *Mycoplasmas* (Tardy et al., 2015; Dordet-Frisoni et al., 2019). A. Maigret et al. found ICEs of 27–30 kb. in one or two copies in seven of the 12 *M. hominis* strains sequenced. However, only five of these ICEs seemed to be functional, as assessed by detection of circular forms of extrachromosomal ICE (Meygret et al., 2019). It was shown that all ICEs found in *M. hominis* had a very similar structure, representing a module of five to six specific to *M. hominis* genes. We found a similar module in one of the isolates, MHO1862. It also contains the tetracycline resistance ribosomal protection protein Tet(M). The location of ICEs and Tet(M) in the same genome may indicate that ICEs are involved in tetracycline resistance. However, the proportion of ICEs was shown to be not higher in isolates carrying the tet(M) gene, suggesting that ICEs are not involved in tetracycline resistance (Maigret et al., 2019). The prevalence of MGEs in clinical strains suggests that they may provide a selective advantage in terms of the physiology or pathogenicity of *M. hominis.*

Comparing the phenotype and proteogenomic profile of 8 clinical isolates and the laboratory strain MHOH34 *M. hominis*, we found that they can be divided into two main groups. The first group consists of isolates MHO7, MHO12, and the laboratory strain MHOH34, all forming TCs, having a relatively high growth rate, significant genome sequence homology, protein abundance, composition of RM systems and additional HsdM, HsdS, and HsdR subunits and the composition and quantity of mobile elements. This group stands out and significantly differs from all other isolates, which are characterized by a lower growth rate and reduced colony size. Within the second group, there is also clustering. Analogous to genome sequence, protein abundance, composition of RM systems, the number of mobile elements, and the Vaa sequence, isolates MHO43 and MHO11, MHO40 and MHO45, MHO1862 and MHO33 can be grouped.

## 4 Conclusion

Thus, based on the comparative proteogenomic analysis of the laboratory strain and eight clinical isolates, we suggest that residing in the host organism *Mycoplasma* has to adapt to a competitive and unfavorable environment. Therefore, it undergoes phenotypic restructuring leading to the slowdown of main cellular processes. This is associated with the switching of carbon metabolism and the activation of the most energetically unfavorable pathway, namely the nucleoside utilization pathway. We propose that DNA methylation may play a role in regulating this switch. We also show that the presence of ICEs in the genomes of clinical isolates may be important for selective advantage in the host. We have shown that proteogenomic analysis makes it possible to determine the phenotype of the *M. hominis* isolate, clearly separating the phenotype that forms TCs and has a high growth rate from the persistent phenotype that forms aTCs.

## Data availability statement

The datasets presented in this study can be found in online repositories. The names of the repository/repositories and accession number(s) can be found in the article/**Supplementary Material**.

## Ethics statement

This study uses strains obtained from The Research Institute of Obstetrics, Gynecology and Reproductology, St Petersburg, Russia. Ethics Committee of the Federal State Budgetary Institution Lopukhin Federal Research and Clinical Center of Physical-chemical Medicine FMBA of Russia (created by Order No. 9 of February 13, 2012) did not require the study to be reviewed or approved by an ethics committee for the studies on humans because bacterial clinical strains were gifted from another research group (The Research Institute of Obstetrics, Gynecology and Reproductology, St Petersburg). The studies were conducted in accordance with the local legislation and institutional requirements. Written informed consent for participation was not required from the participants or the participants’ legal guardians/next of kin in accordance with the national legislation and institutional requirements. The human samples used in this study were acquired from gifted from another research group.

## Author contributions

OP: Conceptualization, Data curation, Investigation, Methodology, Validation, Visualization, Writing – original draft, Writing – review & editing. MG: Conceptualization, Formal analysis, Investigation, Methodology, Validation, Writing – review & editing, Writing – original draft. KS: Conceptualization, Data curation, Investigation, Methodology, Validation, Visualization, Writing – original draft, Writing – review & editing. DU: Data curation, Methodology, Writing – review & editing. AA: Data curation, Methodology, Writing – review & editing. MM: Data curation, Methodology, Writing – review & editing. VB: Data curation, Methodology, Writing – review & editing. IS: Data curation, Methodology, Writing – review & editing. AG: Conceptualization, Data curation, Funding acquisition, Investigation, Methodology, Writing – original draft, Writing – review & editing.
