# Supplementary material for: Unraveling the adaptive strategies of Mycoplasma hominis through proteogenomic profiling of clinical isolates
Source: Front Cell Infect Microbiol. 2024 May 2;14:1398706. doi: 10.3389/fcimb.2024.1398706 (PMC11096450; doi:10.3389/fcimb.2024.1398706)
Supplement: Supplementary file 1 [file DataSheet_1.pdf]

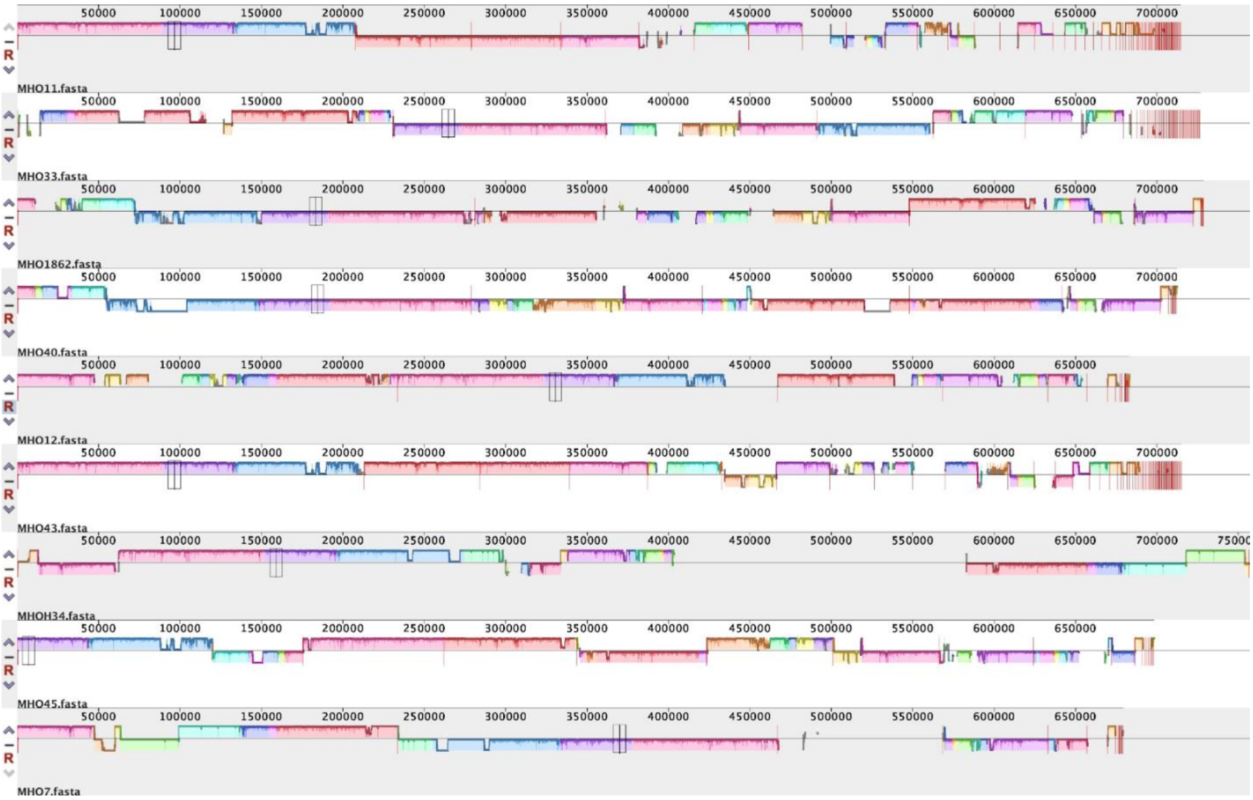

Figure S1. Multiple alignment of the genomes of the laboratory strain MHOH34 and clinical isolates MHO7, MHO45, MHO43, MHO12, MHO40, MHO1862, MHO33 and MHO11 *M hominis*.

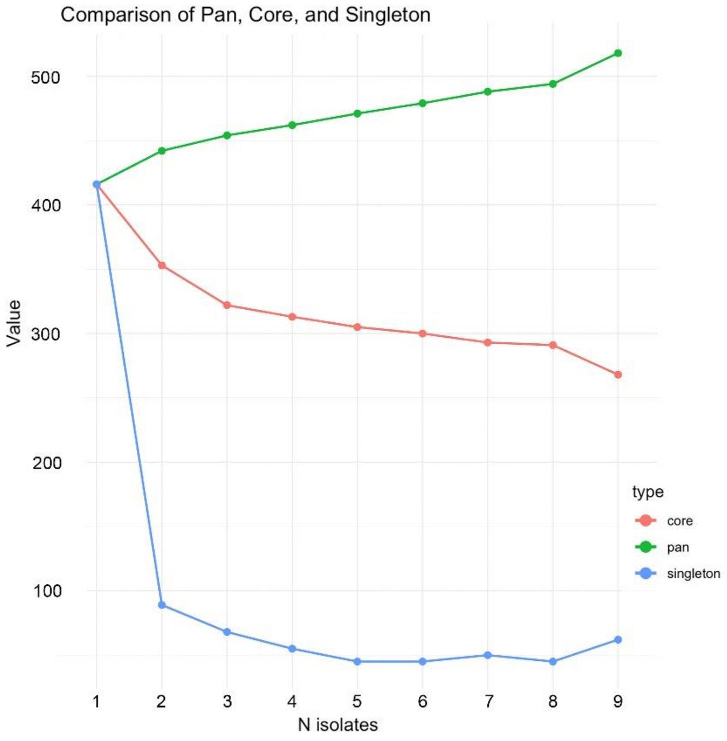

Figure S2. Gene number estimation curves for the eight clinic isolates MHO7, MHO45, MHO43, MHO12, MHO40, MHO1862, MHO33 and MHO11 and in laboratory strain MHOH34 core genome and core proteome (red and yellow curve), pan-genomes and pan-proteome (green and emerald curve).

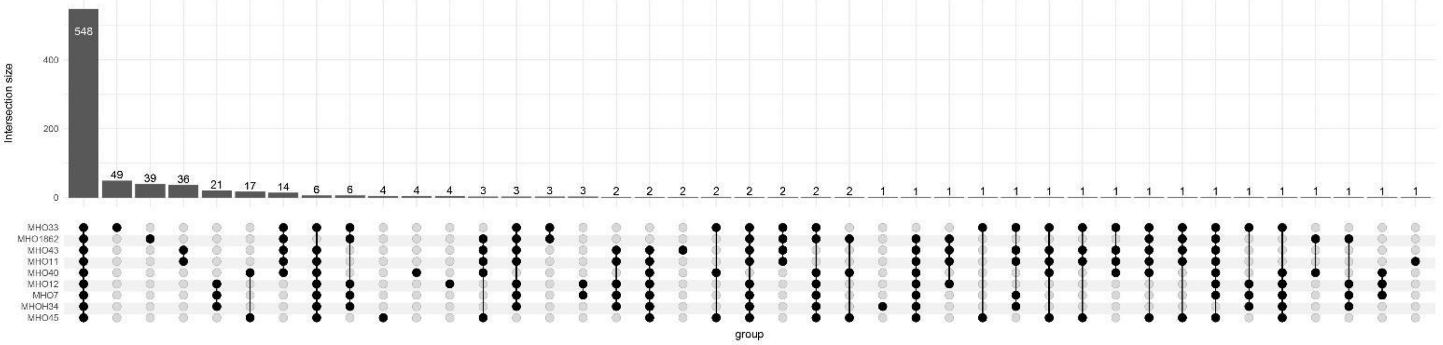

Figure S3. Upset plot showing the number of common and unique protein-coding genes for the laboratory strain MHOH34 and clinical isolates MHO7, MHO45, MHO43, MHO12, MHO40, MHO1862, MHO33 and MHO11 *M. hominis*.

Table S1. Amino acid substitutions in GyrA, GyrB, ParE, ParC and TopA of laboratory strain MHOH34 and clinical isolates MHO7, MHO11, MHO12, MHO33, MHO40, MHO43, MHO45 and MHO1862 *M. hominis.*

**Colony type**	**TC**	**TC**	**TC**	**aTC**	**aTC**	**aTC**	**aTC**	**aTC**	**aTC**	
**Protein (size)\Strain**	**MHOH34**	**MHO12**	**MHO7**	**MHO33**	**MHO45**	**MHO43**	**MHO40**	**MHO1862**	**MHO11**	**SAP position**
**GyrA (911)**	K	K	K	K	K	R	K	K	R	79
	V	V	V	V	V	V	L	V	V	98
	E	E	E	E	E	K	E	E	K	272
	D	D	D	D	N	D	N	D	D	745
	L	L	L	I	I	I	I	L	I	764
**GyrB (648)**	A	A	A	A	A	S	A	A	S	302
	I	I	I	I	T	T	T	T	T	313
	I	I	I	I	I	V	I	I	V	385
**ParE (639)**	D	D	D	D	D	D	D	N	D	209
	V	V	V	A	A	A	A	A	A	317
**ParC (933)**	K	K	K	K	R	K	R	R	K	144
	A	A	A	V	A	A	A	A	A	154
	L	L	L	L	L	F	L	L	F	484
	S	S	S	N	N	N	S	S	N	542
	H	H	H	H	R	H	H	H	H	579
	K	K	K	K	K	K	R	K	K	582
	I	I	I	M	I	I	I	M	I	623
	I	I	I	V	V	V	V	V	V	632

	I	I	I	V	V	V	V	V	V	718
	T	T	T	T	T	T	A	A	T	738
	D	D	D	N	D	D	N	D	D	828
	N	N	N	N	N	N	N	D	N	861
	Q	Q	Q	E	Q	Q	Q	Q	Q	862
	G	G	G	D	D	D	D	D	D	894
	D	D	D	D	D	N	N	D	N	898
	V	V	V	V	V	I	V	V	I	908
**TopA (612)**	R	R	R	K	R	R	R	R	R	141
	V	V	V	I	I	V	I	I	V	190
	D	D	D	D	D	D	D	Y	D	332
	S	S	S	S	S	S	S	T	S	347
	S	S	S	S	S	S	S	F	S	416
	S	S	S	S	S	L	S	S	S	463
	V	V	V	V	V	V	F	V	V	494
	I	I	I	V	I	V	I	I	V	530

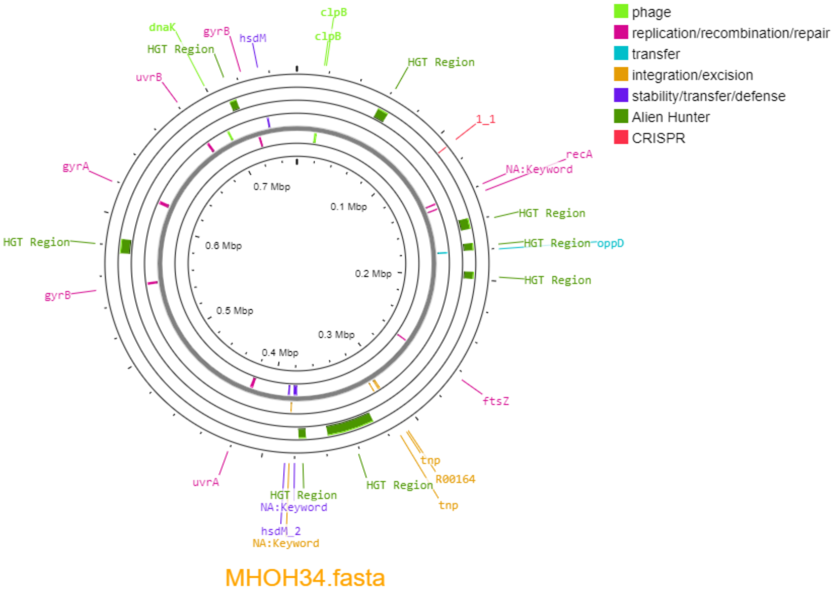

Figure S4, A. Analysis of mobile elements using mobile OG, representing key subdivisions of the molecular machinery associated with MGEs: replication/recombination/repair (RRR), integration/excision (IE), stability/transfer/defense (STD), interorganismal transfer (T) and phage (P) and possible horizontal gene transfer (HTG) events (Alien Hunter) in the genomes of laboratory strain MHOH34 of *M. hominis.*

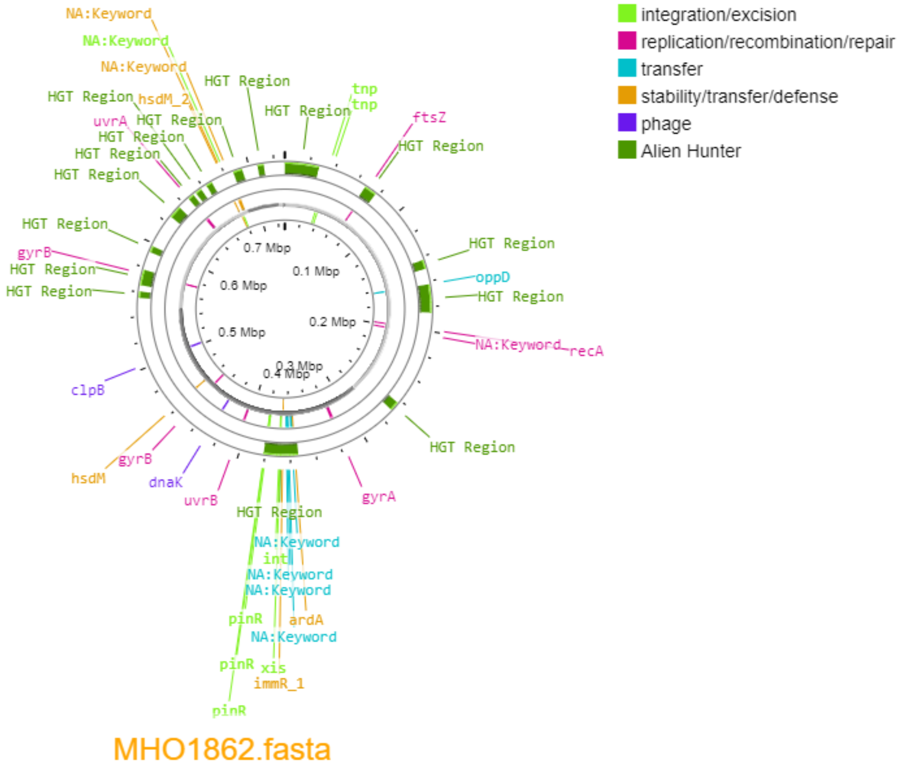

Figure S4, B. Analysis of mobile elements using mobile OG, representing key subdivisions of the molecular machinery associated with MGEs: replication/recombination/repair (RRR), integration/excision (IE), stability/transfer/defense (STD), interorganismal transfer (T) and phage (P) and possible horizontal gene transfer (HTG) events (Alien Hunter) in the genomes of the clinic isolate MHOH34 of *M. hominis*

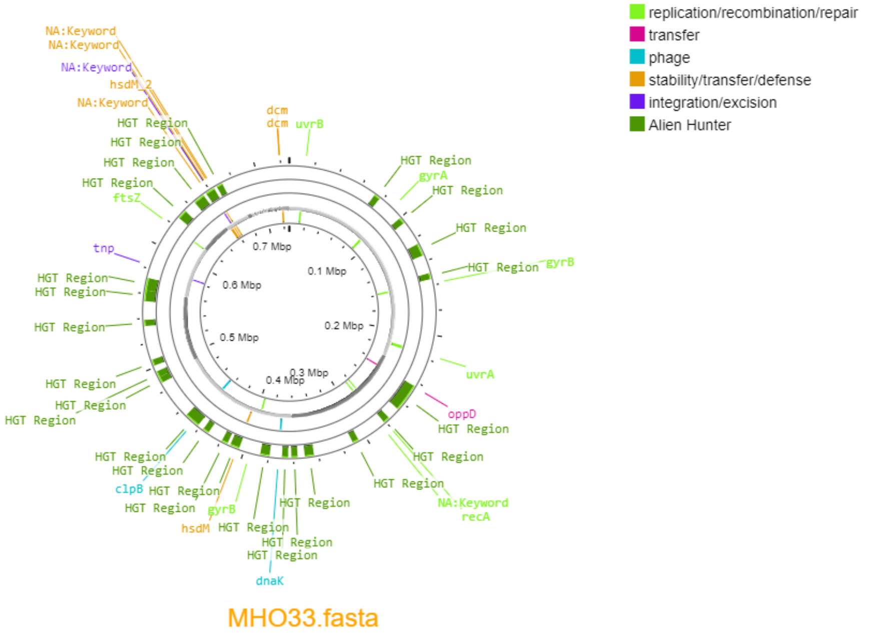

Figure S4, C. Analysis of mobile elements using mobile OG, representing key subdivisions of the molecular machinery associated with MGEs: replication/recombination/repair (RRR), integration/excision (IE), stability/transfer/defense (STD), interorganismal transfer (T) and phage (P) and possible horizontal gene transfer (HTG) events (Alien Hunter) in the genomes of the clinic isolate MHO33 of *M. hominis*

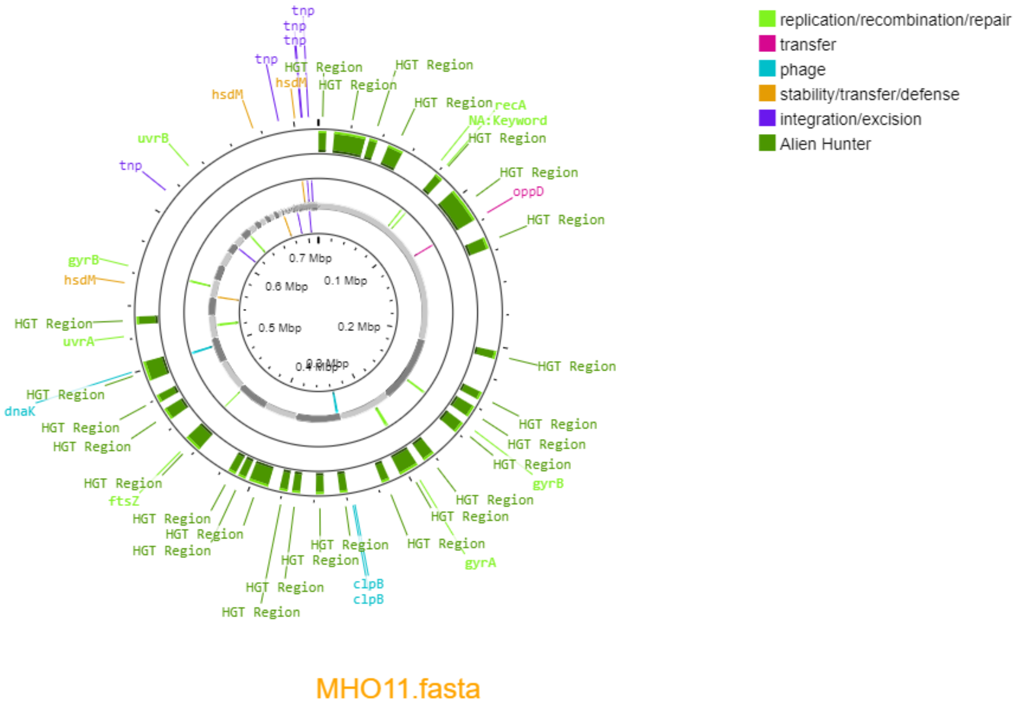

Figure S4, D. Analysis of mobile elements using mobile OG, representing key subdivisions of the molecular machinery associated with MGE: replication/recombination/repair (RRR), integration/excision (IE), stability/transfer/defense (STD), interorganismal transfer (T) and phage (P) and possible horizontal gene transfer (HTG) events (Alien Hunter) in the genomes of the clinic isolate MHO11 *M. hominis*.

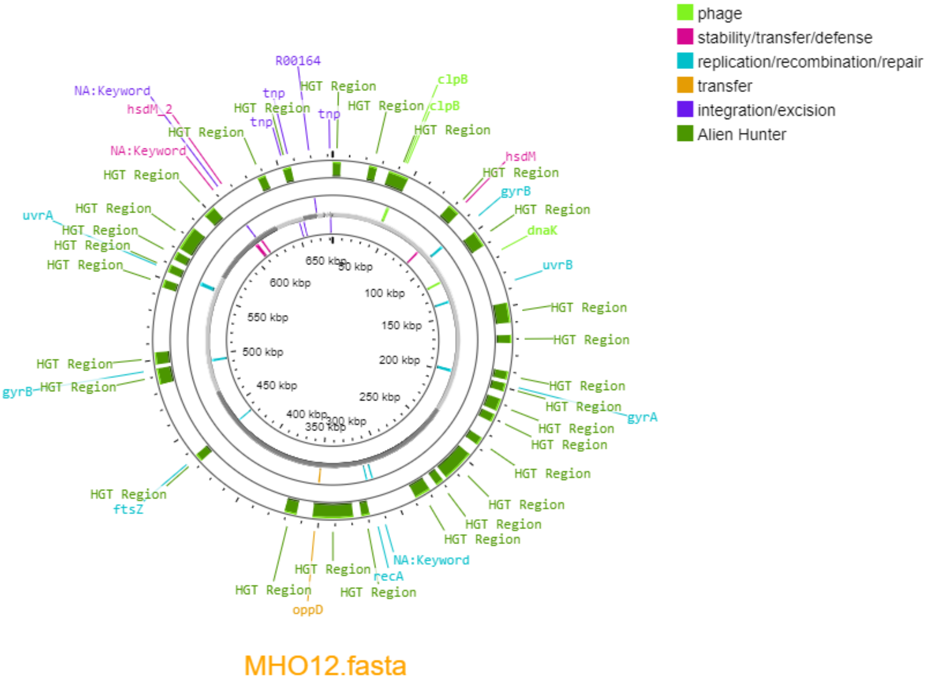

Figure S4, E. Analysis of mobile elements using mobile OG, representing key subdivisions of the molecular machinery associated with MGEs: replication/recombination/repair (RRR), integration/excision (IE), stability/transfer/defense (STD), interorganismal transfer (T) and phage (P) and possible horizontal gene transfer (HTG) events (Alien Hunter) in the genomes of the clinic isolate MHO12 of *M. hominis*.

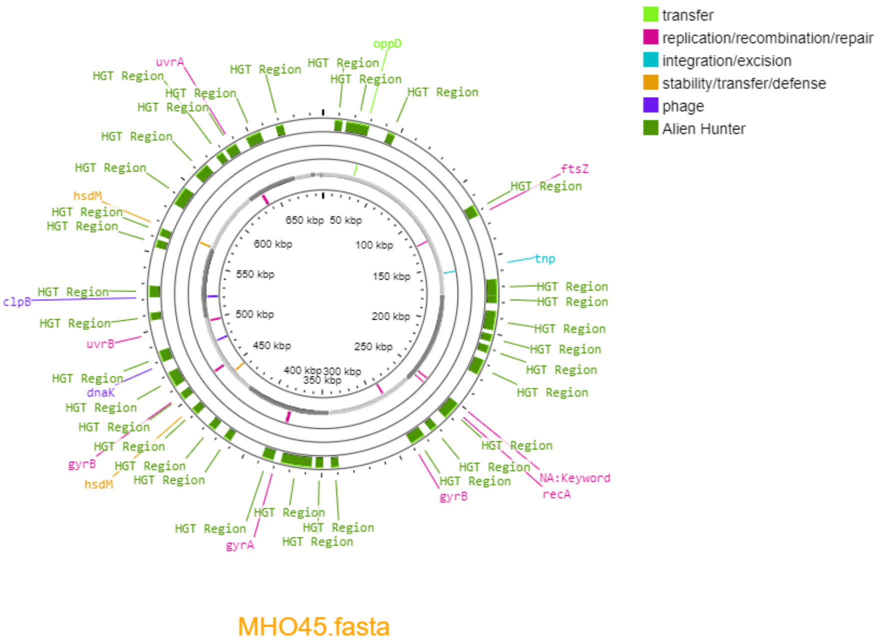

Figure S4, F. Analysis of mobile elements using mobile OG, representing key subdivisions of the molecular machinery associated with MGEs: replication/recombination/repair (RRR), integration/excision (IE), stability/transfer/defense (STD), interorganismal transfer (T) and phage (P) and possible horizontal gene transfer (HTG) events (Alien Hunter) in the genomes of the clinic isolate MHO45 of *M. hominis*.

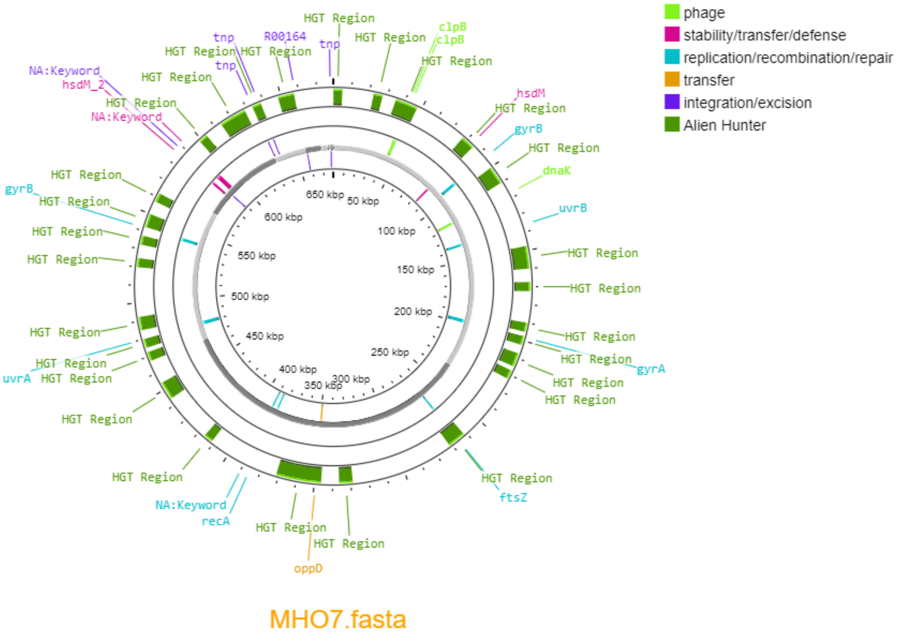

Figure S4, G. Analysis of mobile elements using mobile OG, representing key subdivisions of the molecular machinery associated with MGEs: replication/recombination/repair (RRR), integration/excision (IE), stability/transfer/defense (STD), interorganismal transfer (T) and phage (P) and possible horizontal gene transfer (HTG) events (Alien Hunter) in the genomes of the clinic isolate MHO7 of *M. hominis*.

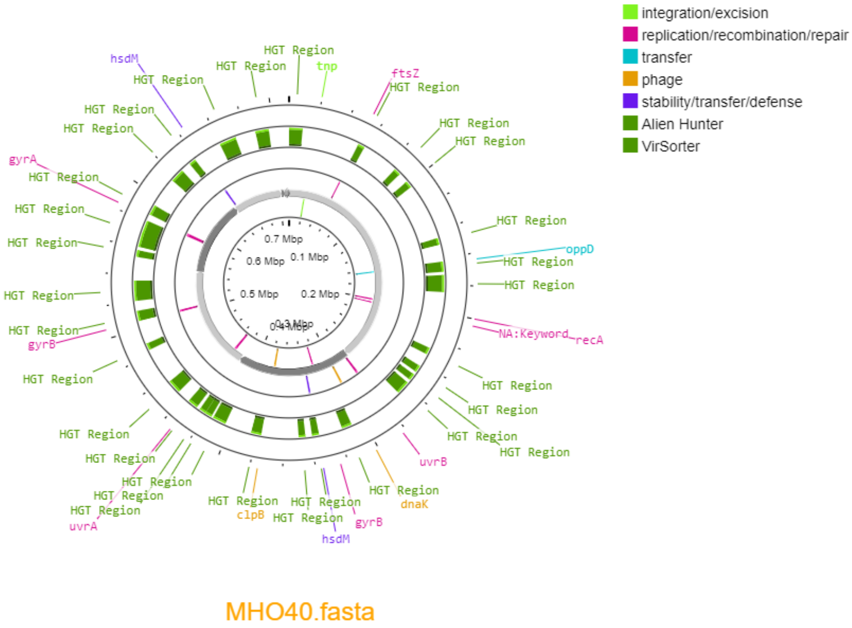

Figure S4, H. Analysis of mobile elements using mobile OG, representing key subdivisions of the molecular machinery associated with MGEs: replication/recombination/repair (RRR), integration/excision (IE), stability/transfer/defense (STD), interorganismal transfer (T) and phage (P) and possible horizontal gene transfer (HTG) events (Alien Hunter) in the genomes of the clinic isolate MHO40 of *M. hominis*.

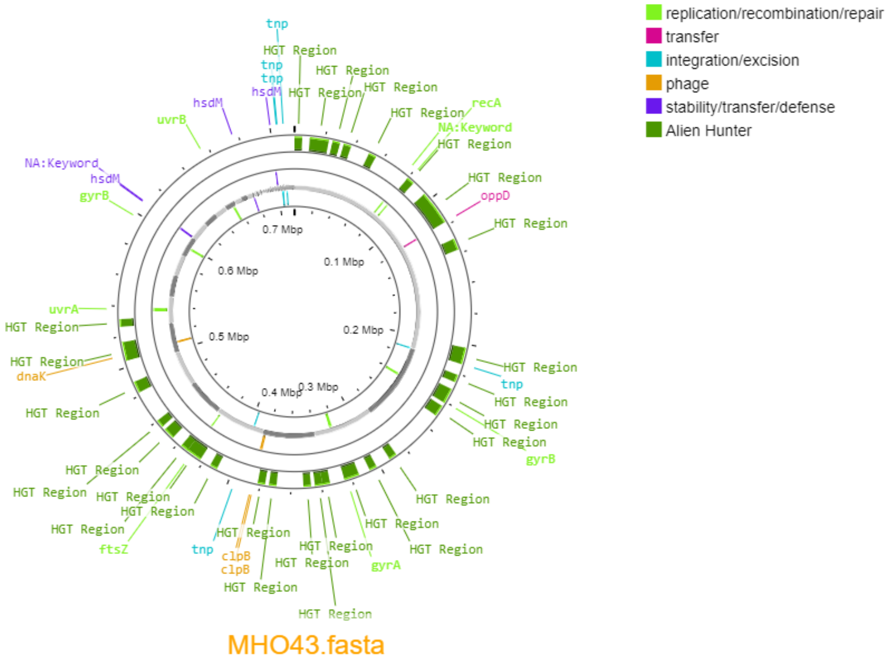

Figure S4, L. Analysis of mobile elements using mobile OG, representing key subdivisions of the molecular machinery associated with MGEs: replication/recombination/repair (RRR), integration/excision (IE), stability/transfer/defense (STD), interorganismal transfer (T) and phage (P) and possible horizontal gene transfer (HTG) events (Alien Hunter) in the genomes of the clinical isolates MHO43 of *M. hominis*.

Table S2. List and number of mobile genetic elements (MGEs) identified in the laboratory strain MHOH34 and in clinical isolates MHO7, MHO45, MHO43, MHO12, MHO40, MHO1862, MHO33 and MHO11 of *M. hominis* using the Proksee program and genome annotation.

**MGEs**	**MHO H34**	**MHO 12**	**MH O 7**	**MHO 33**	**MHO 40**	**MHO 43**	**MHO 45**	**MHO 11**	**MHO 1862**
**MGEs are determined according to the Proksee**									
mobileOG_000342736	1	1	1						
mobileOG_000318223 tnp transposase	2	4	3	1	1	5	1	5	2
mobileOG_000751459	1	1	1	2					1
mobileOG_000720720 integrase	1	1	1	1					1
mobileOG_000369174|hsdM_2	1	1	1	1					1
mobileOG_000167331|hsdM	1	1	1	2	2	1	2	1	1
mobileOG_000233664|hsdM						1		1	
mobileOG_000143174|hsdM						1		1	
mobileOG_000727300				1					
mobileOG_000174181|dcm DNA-cytosine methyltransferase				1					
mobileOG_000546653 restriction endonuclease subunit S						1			
mobileOG_000033447|ardA antirestriction protein									1

mobileOG_000720570 conjugative transposon protein									1
mobileOG_000720567 conjugative transposon membrane protein									1
mobileOG_000403593 peptidoglycan hydrolase-conjugation iap									1
mobileOG_000720204 conjugal transfer protein									1
mobileOG_000267388|immR_1									1
mobileOG_000269634|xis ICEBs1 excisionase									1
mobileOG_000004240|int integrator complex subunit 1									1
mobileOG_000267349|pinR serine recombinase									1
mobileOG_000269846|pinR serine recombinase									1
mobileOG_000267352|pinR serine recombinase									1
mobileOG_000727300									
**MGEs are determined according to the annotation**									
Truncated integrase-recombinase protein					1				
Terminase-6 domain-containing protein				1	1	1		1	1
Htpn Mycoplasma phage MAV1					1			1	1
Type II CRISPR RNA-guided endonuclease Cas9				1	1	1		1	1
Phage protein				1	1		1	1	1
Major capsid protein				1	1	1		1	1
Tyr recombinase domain-containing protein		2	2	1	1	1		1	3
DJ-1/PfpI family protein		1	1	1	1		1	1	1
Phg-2220-C domain-containing protein		1	1						
Phage-int-SAM-5 domain-containing protein			1						
Holliday junction resolvase RecU		1	1	1		1	1	1	
ComEC/Rec2 family competence protein		1	1	1		1		1	
Integrase-SAM-like-N domain-containing protein		1		1		1		1	
replication-associated recombination protein A		1		1		1	1		1
Holliday junction resolvase RuvX		1		1			1	1	1
DDE-type integrase/transposase/recombinase				1					
Holliday junction branch migration DNA helicase RuvB		1	1	1			1		1

Holliday junction branch migration protein RuvA		1	1	1			1		1
Type IV secretory pathway, VirB4 component									1
Conjugative transposon protein TcpC									1
Tetracycline resistance ribosomal protection protein Tet(M)									1
START domain-containing protein							1		1

Table S3. Complete list of type I restriction-modification system subunits identified in the genomes of the laboratory strain MHOH34 and clinical isolates MHO7, MHO45, MHO43, MHO12, MHO40, MHO1862, MHO33 and MHO11 of *M. hominis*.

ID	protein	MHOH34	MHO7	MHO12	MHO33	MHO40	MHO43	MHO45	MHO11	MHO1862	Start	Stop	contig
MHO1862_02125	Restriction endonuclease subunit S									1	181691	184072	contig_2
MHO1862_02130	type I restriction endonuclease subunit R									1	184104	187187	contig_2
MHO1862_02945 MHO33_02965	restriction endonuclease subunit S				1					1	130995	132116	contig_3
MHO33_00050	Type-2 restriction enzyme				1						8023	8898	contig_1
MHO33_01785	Restriction endonuclease subunit S				1						44654	47071	contig_3
MHO33_02970, MHOH34_01850 MHO7_02630 MHO12_02785	restriction endonuclease subunit S	1	1	1	1						7120	8187	contig_7
MHO33_02975 MHO1862_02940	restriction endonuclease subunit S				1					1	8195	8617	contig_8
MHO33_03050	Site-specific DNA-methyltransferase (adenine-specific)				1						1464	3803	contig_8
MHO33_03350	DNA (cytosine-5-)-methyltransferase				1						1	351	contig_51
MHO33_03355	DNA (cytosine-5-)-methyltransferase				1						1	351	contig_52
MHO40_01555 MHO45_01945	Restriction endonuclease subunit S					1		1			56880	59381	contig_2
MHO40_01710	Site-specific DNA-methyltransferase					1					89895	91091	contig_2
MHO40_02000 MHO1862_02760 , MHO45_02835	N6-Mtase domain-containing protein					1		1		1	21011	22606	contig_3
MHO40_02770, MHO43_02755, MHO11_02500, 3MHO45_02450	type I restriction-modification system subunit M					1	1	1	1		899	2461	contig_5
MHO43_01935, MHO11_01860	Type I restriction endonuclease subunit M						1		1		3	275	contig_6
MHO43_01940, MHO11_01850	Restriction endonuclease subunit S						1		1		278	2098	contig_6

MHO43_01940, MHO11_01855	Restriction endonuclease subunit S						1		1		278	2098	contig_6
MHO43_01945, MHO11_01845	restriction endonuclease subunit S						1		1		2076	2690	contig_6
MHO43_02095, MHO11_01695	Site-specific DNA-methyltransferase						1		1		30525	31658	contig_6
MHO43_02345, MHO11_02275	Cytosine methyltransferase						1		1		10249	10923	contig_8
MHO43_02410, MHO11_02210	Site-specific DNA-methyltransferase (adenine-specific)						1		1		26075	26833	contig_8
MHO43_02985, MHO11_02975	site-specific DNA-methyltransferase (adenine-specific)						1		1		1	783	contig_20
MHO43_03230, MHO11_03230	Type I restriction endonuclease subunit M						1		1		47	319	contig_59
MHO45_02150	Site-specific DNA-methyltransferase (adenine-specific)							1			71424	72272	contig_5
MHO45_02155	Type-2 restriction enzyme							1			72263	73138	contig_5
MHOH34_03040, MHO7_00495, MHO33_01645, MHO12_00495	Cytosine methyltransferase	1	1	1	1						118371	119207	contig_1
MHOH34_00075, MHO7_00190, MHO12_00190	Type III restriction-modification system methylase	1	1	1							11132	11746	contig_1
MHOH34_00295, MHO7_02115, MHO12_00995	N-6 DNA methylase	1	1	1							70087	71502	contig_1
MHOH34_01495, MHO7_02875, MHO12_02985	Restriction endonuclease	1	1	1							310355	311059	contig_1
MHOH34_01500, MHO7_02880, MHO12_02980	DNA (cytosine-5-)-methyltransferase	1	1	1							311040	312602	contig_1
MHOH34_01845, MHO7_02635, MHO33_02950, MHO12_02780, MHO1862_02960	type I restriction endonuclease subunit R	1	1	1	1					1	378481	381582	contig_1
MHOH34_01855, MHO7_02625, MHO33_02955, MHO12_02790, MHO1862_02955	restriction endonuclease subunit S	1	1	1	1					1	382426	382839	contig_1
MHOH34_01865, MHO7_02615, MHO12_02800	restriction endonuclease subunit S	1	1	1							382875	383675	contig_1
MHOH34_01870, MHO7_02610, MHO12_02805	restriction endonuclease subunit S	1	1	1							384698	385210	contig_1
MHOH34_01875, MHO7_02605, MHO33_02980, MHO12_02810, MHO1862_02935	type I restriction-modification system subunit M	1	1	1	1					1	385179	386660	contig_1
MHOH34_03180, MHO40_01550, MHO7_00355, MHO43_03155, MHO11_03155, MHO33_01780, MHO12_00355, MHO1862_02120, MHO45_01950	type I restriction-modification system subunit M	1	1	1	1	1	1	1	1	1	732745	734307	contig_1

MHOH34_03185, MHO7_00350, MHO12_00350	restriction endonuclease subunit S	1	1	1							734310	736853	contig_1
MHOH34_03190, MHO40_01560, MHO7_00345, MHO43_01950, MHO11_01840, MHO33_01790, MHO12_00345, MHO45_01940	type I restriction endonuclease subunit R	1	1	1	1	1	1	1	1		736885	739968	contig_12
MHO11_02575	restriction endonuclease subunit S								1		1	756	contig_54
MHO11_03175, MHO43_03175	restriction endonuclease subunit S						1		1		1	486	contig_13
MHO43_02760	restriction endonuclease subunit S						1		1		1	153	contig_1
MHO43_02765, MHO11_02580	restriction endonuclease subunit S										131	1345	contig_13
MHOH34_01835, MHO7_02645, MHO12_02770	5-methylcytosine-specific restriction endonuclease system specificity protein McrC	1	1	1							374286	375284	contig_1

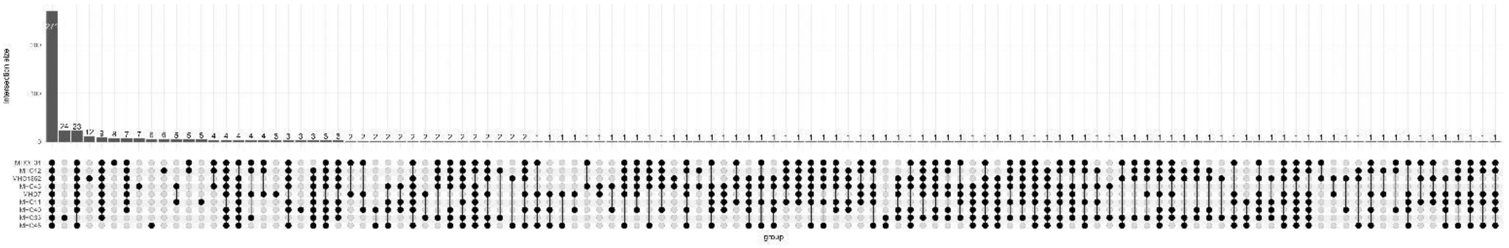

Figure S5. Upset plot showing the number of common and unique protein-coding genes for the laboratory strain MHOH34 and clinical isolates MHO7, MHO45, MHO43, MHO12, MHO40, MHO1862, MHO33 и MHO11 of *M hominis*.

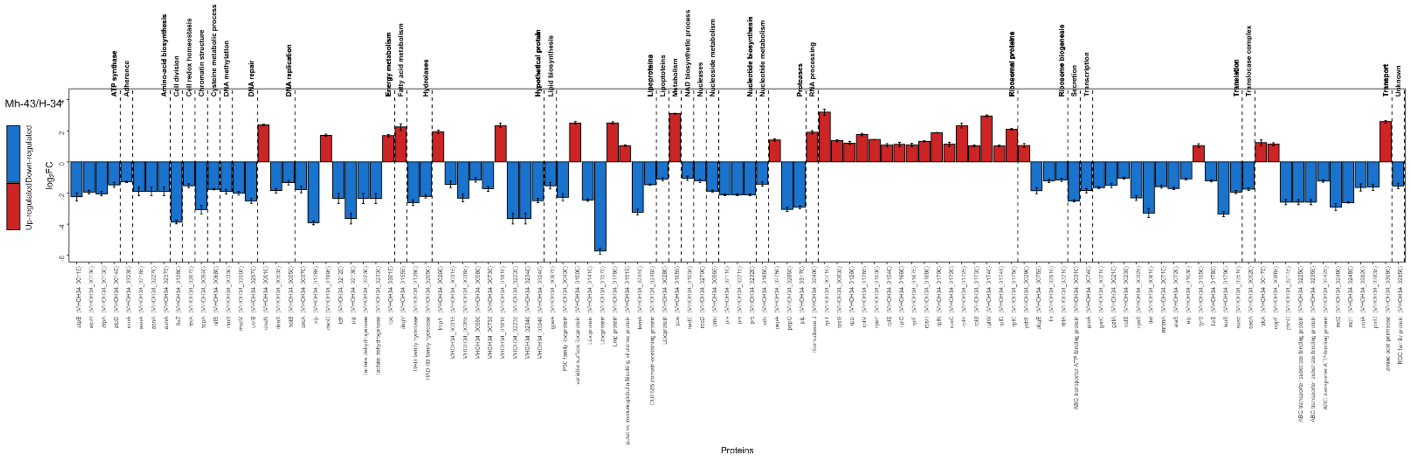

Figure S6, A. Functional groups of differentially expressed proteins of clinical isolates MHO43 compared with laboratory strain MHOH34 *M. hominis.*

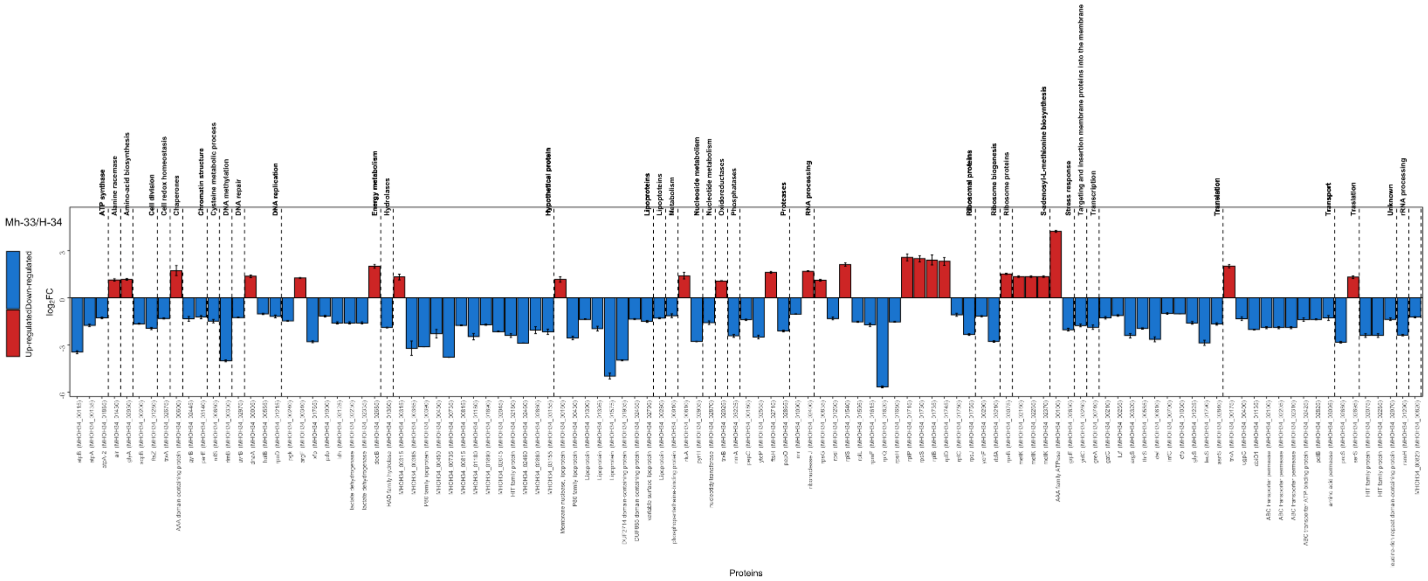

Figure S6, B. Functional groups of differentially expressed proteins of clinical isolates MHO33 compared with laboratory strain MHOH34 *M. hominis.*

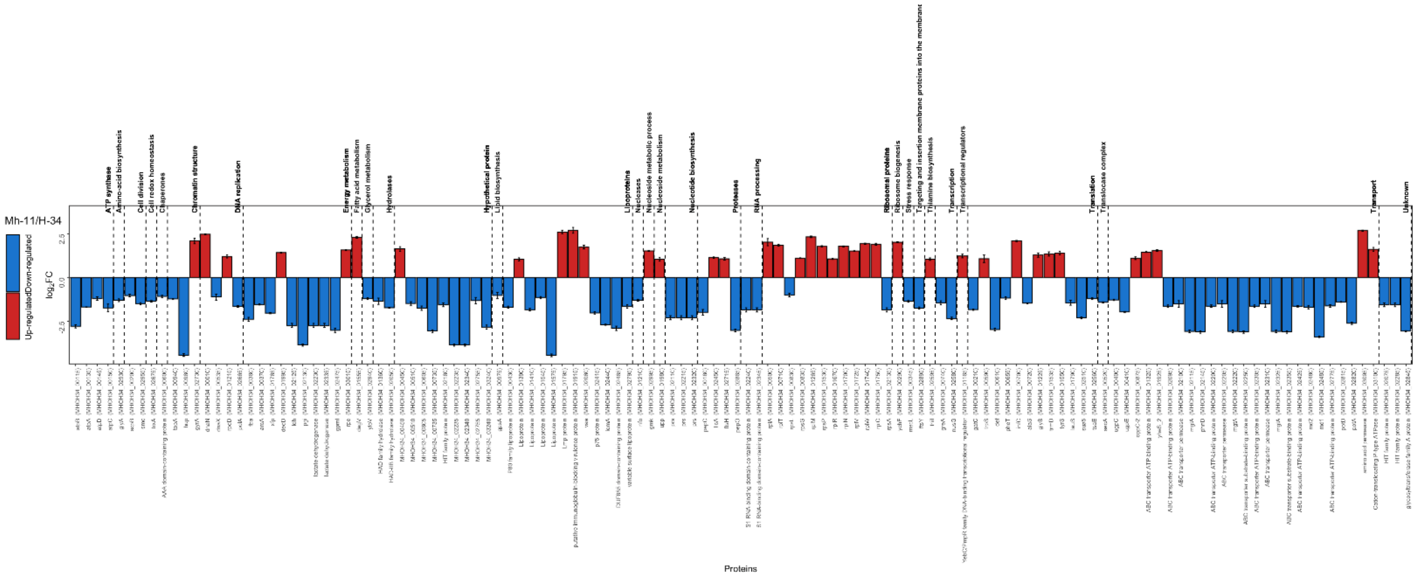

Figure S6, C. Functional groups of differentially expressed proteins of clinical isolates MHO11 compared with laboratory strain MHOH34 *M. hominis.*

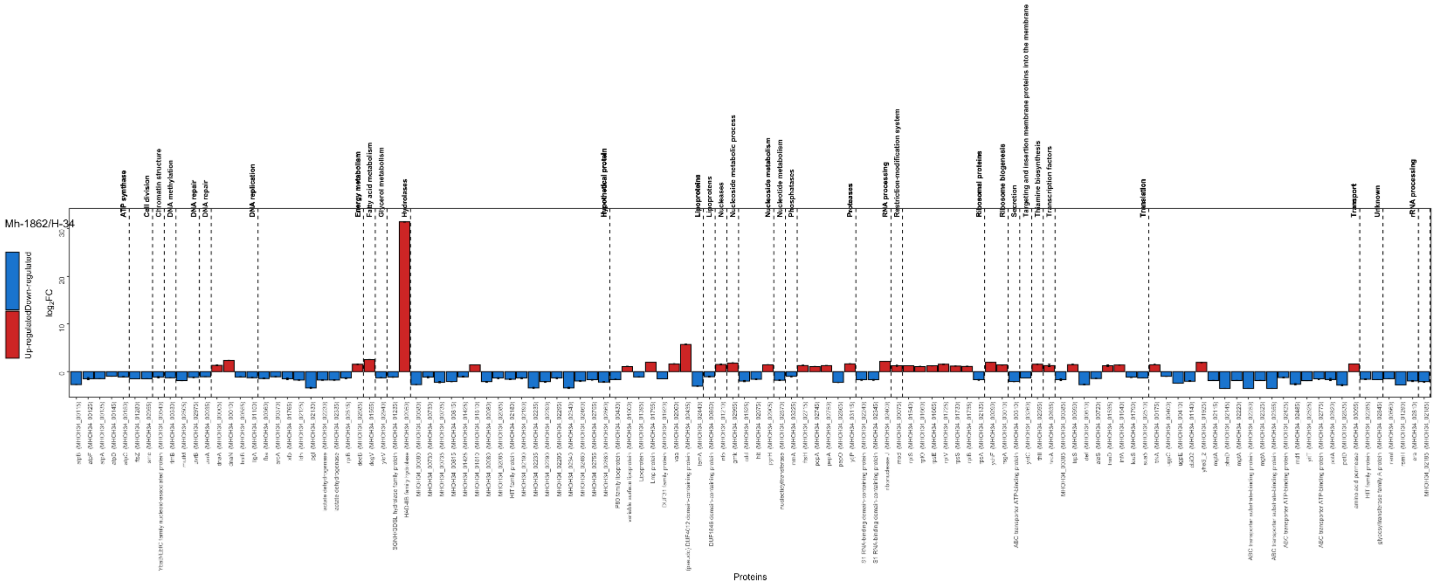

Figure S6, D. Functional groups of differentially expressed proteins of clinical isolates MHO1862 compared with laboratory strain MHOH34 *M. hominis.*

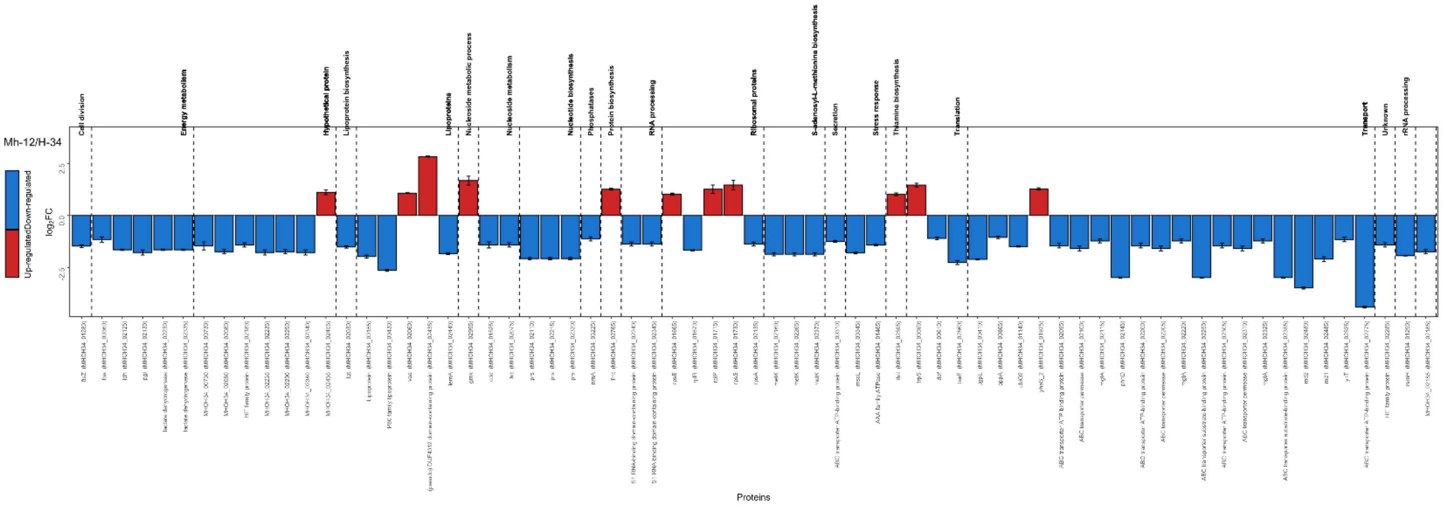

Figure S6, E. Functional groups of differentially expressed proteins of clinical isolates MHO12 compared with laboratory strain MHOH34 *M. hominis*.

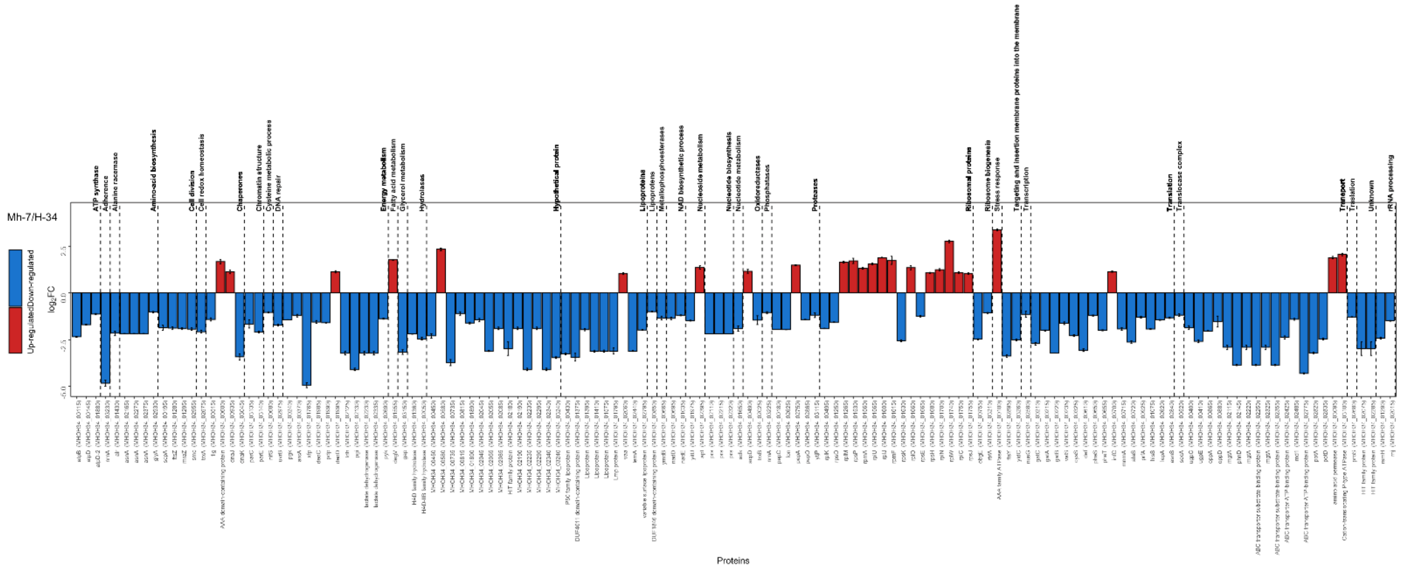

Figure S6, F. Functional groups of differentially expressed proteins of clinical isolates MHO7 compared with laboratory strain MHOH34 *M. hominis*.

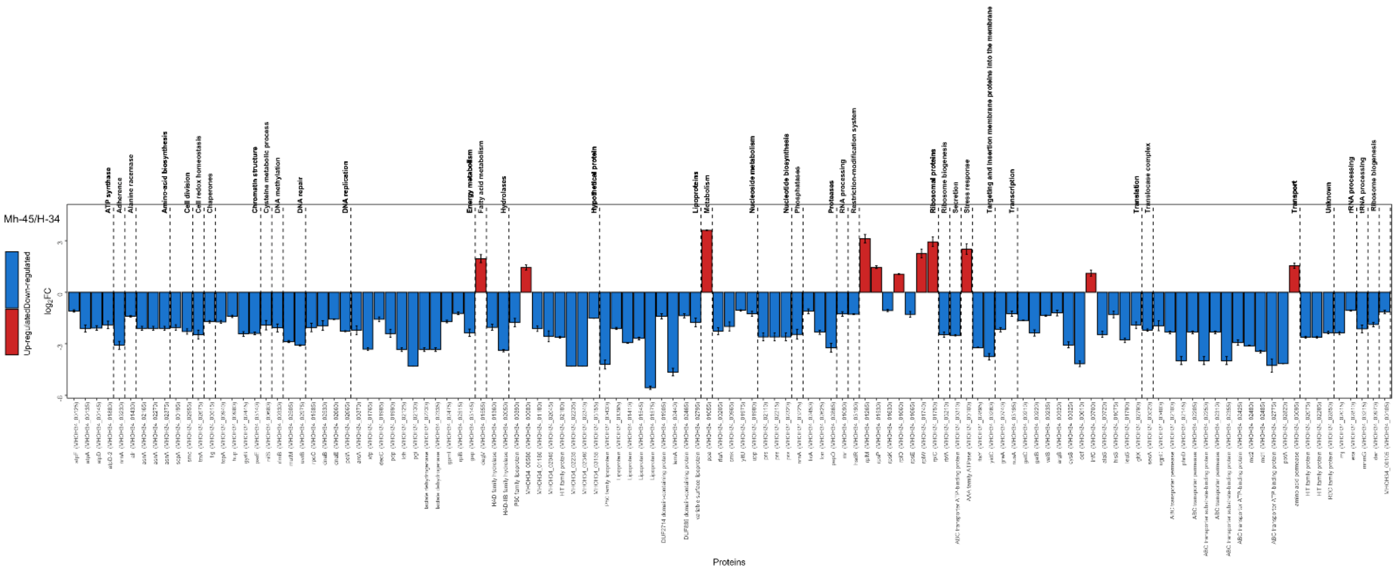

Figure S6, G. Functional groups of differentially expressed proteins of clinical isolates MHO45 compared with laboratory strain MHOH34 *M. hominis.*

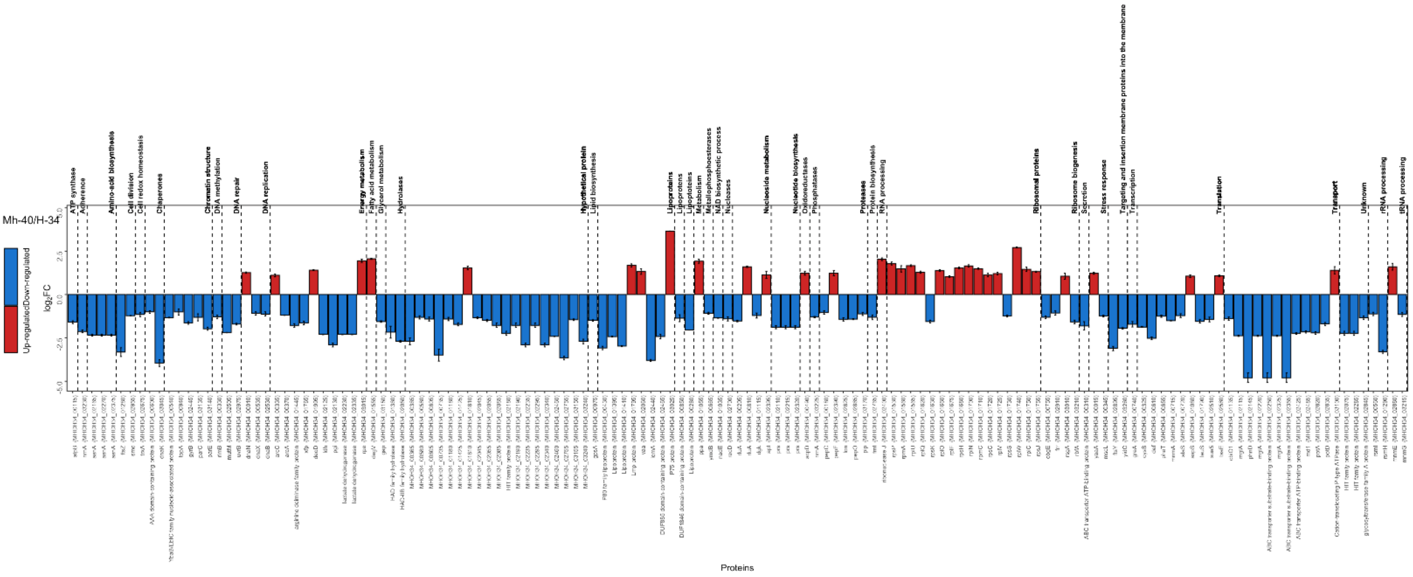

Figure S6, H. Functional groups of differentially expressed proteins of clinical isolates MHO40 compared with laboratory strain MHOH34 *M. hominis.*

Table S5. Amino acid substitutions in energy metabolism enzymes of laboratory strain MHOH34 and clinical isolates MHO7, MHO11, MHO12, MHO33, MHO40, MHO43, MHO45 and MHO1862 *M. hominis*.

	Colony type	TC	TC	TC	aTC	aTC	aTC	aTC	aTC	aTC	
**Metabolic pathway**	**Protein (size)\Strain**	**MHOH 34**	**MHO 12**	**MHO 7**	**MHO 33**	**MHO 45**	**MHO 43**	**MHO 40**	**MHO 1862**	**MHO 11**	**SAP position**
**Arginine deiminase pathway**	**ArcA (411)**	A	A	A	A	S	A	S	A	A	80
		E	E	E	E	E	E	E	D	E	222
		T	T	T	P	P	P	P	P	P	307
	**ArcB (349)**	E	E	E	E	E	E	K	E	E	20
		N	N	N	D	D	D	D	D	D	202
	**ArcC (313)**	F	F	F	Y	Y	Y	Y	Y	Y	235
		N	N	N	D	D	D	D	D	D	247
	**ArcD (-)**	-	-	-	-	-	-	-	-	-	-
	**DDAH (285)**	Q	Q	Q	K	K	K	K	K	K	11
		L	L	L	I	L	L	L	L	L	97
**Glycolysis**	**GAPD (335)**	I	I	I	V	V	V	V	V	V	67
		Y	Y	Y	F	Y	F	Y	F	F	101
		I	I	I	M	I	M	I	M	M	262
		D	D	D	E	D	E	D	E	E	302
	**PGK (399)**	A	A	A	A	A	A	A	T	A	98
	**PGM (499)**	K	K	K	E	E	K	E	E	K	54
		D	D	D	N	D	N	D	D	N	87
		N	N	N	N	N	H	N	N	H	106
		E	E	E	G	G	G	G	G	G	138
		D	D	D	D	D	N	D	D	N	200
		K	K	K	E	K	E	K	K	E	211
		V	V	V	I	I	I	I	I	I	213
		K	K	K	E	E	E	E	E	E	231
		V	V	V	V	A	V	A	A	V	239
		N	N	N	N	S	N	S	S	N	268
		K	K	K	E	K	K	K	K	K	273
		S	S	S	S	S	N	S	S	N	313
		I	I	I	I	V	I	V	V	I	377
		E	E	E	G	E	G	E	E	G	469
	**ENO (457)**	V	V	V	V	I	V	I	V	V	234
		A	A	A	S	S	A	S	S	A	438

	PYK (473)	I	I	I	I	I	V	I	I	V	63
		K	K	K	K	R	K	R	K	K	97
		N	N	N	D	D	D	D	D	D	142
		V	V	V	V	I	V	I	I	V	143
		T	T	T	T	T	I	T	I	I	149
Nucleoside degradation	DeoA (432)	V	V	V	V	V	V	V	I	V	33
		V	V	V	I	I	V	I	I	V	77
		N	N	N	N	N	S	N	S	S	307
		N	N	N	S	S	S	S	S	S	341
		P	P	P	S	P	P	P	P	P	405
	DeoD (238)	Y	Y	Y	F	F	F	F	F	F	188
		K	K	K	R	K	K	K	K	K	149
	DeoB (398)	-	-	-	-	-	-	-	-	-	-
	DeoC (220)	-	-	-	-	-	-	-	-	-	-
Pentose phosphate pathway	Rpe (215)	Q	Q	Q	Q	Q	K	Q	Q	K	104
		R	R	R	R	R	R	R	K	R	126
		A	A	A	A	A	A	A	S	A	161
		D	D	D	D	D	N	D	D	N	168
		N	N	N	N	N	D	N	N	D	193
	RpiB (155)	E	K	K	E	E	E	E	E	E	27
		V	V	V	V	V	V	I	V	V	125
		S	S	S	S	S	S	N	S	S	131
	Xfp, PK (796)	K	K	K	K	K	N	K	K	N	376
		V	V	V	V	A	V	V	V	V	599
		S	S	S	S	S	N	S	S	N	678
	Prs, PRPP (324)	T	K	K	T	T	T	T	T	T	229
		D	D	D	Y	D	D	D	D	D	271
Others	ADH (387)	E	E	E	D	D	D	D	D	D	17
		A	A	A	V	A	A	A	V	A	55
		L	L	L	S	S	L	S	L	L	83
		W	W	W	W	W	R	W	W	R	177
		M	M	M	I	I	M	I	M	M	231
		A	A	A	V	A	A	A	A	A	239
		V	V	V	I	I	V	I	I	V	243
		Q	Q	Q	E	Q	E	Q	E	E	321
		S	S	S	S	S	S	S	G	S	349

	**AK (398)**	V	V	V	V	V	V	I	V	V	171
		R	R	R	K	K	K	K	K	K	293
		D	D	D	D	D	N	D	D	N	297
		T	T	T	T	I	T	T	T	T	397
	**GPDH (327)**	D	D	D	N	D	N	N	N	D	313
	**TPI (243)**	K	K	K	R	K	K	K	K	K	55
	**LDH**	-	-	-	-	-	-	-	-	-	-

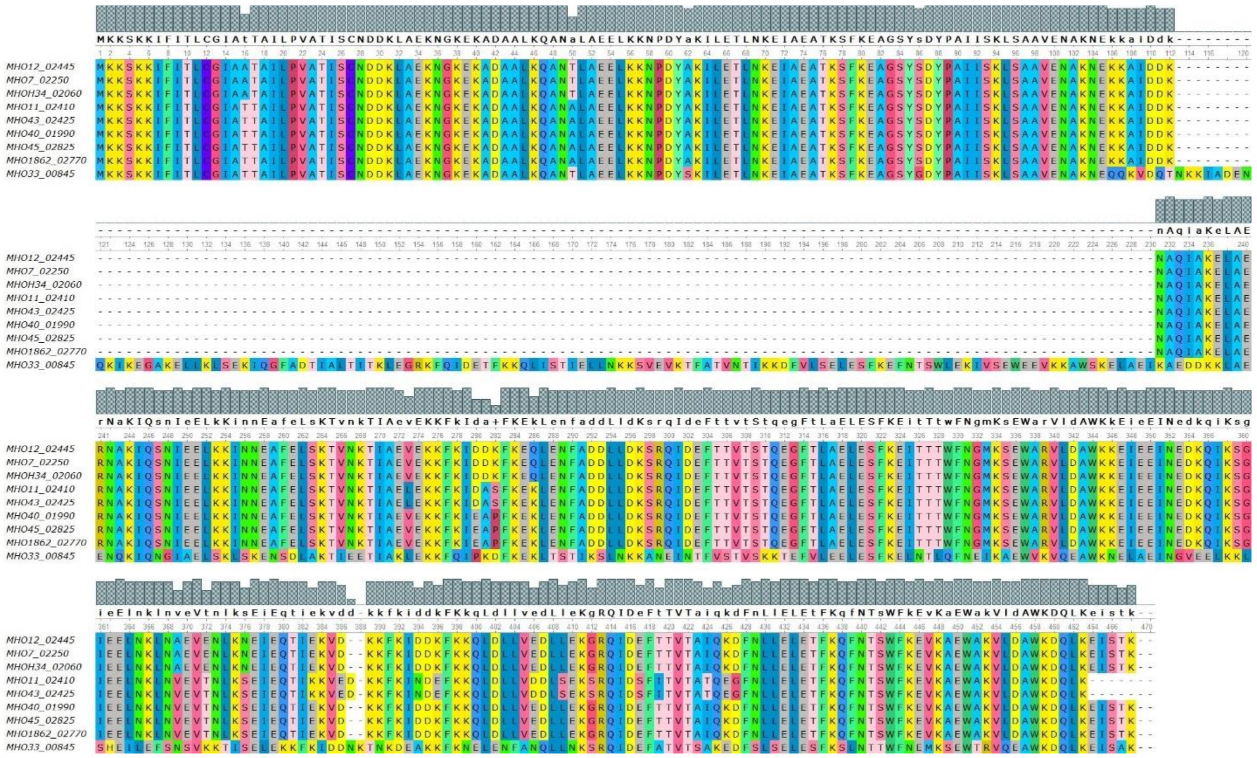

Figure S7. Comparative analysis of the amino acid sequence of the variable Vaa antigen of laboratory strain MHOH34 and clinical isolates MHO7, MHO12, MHO43, MHO45, MHO40, MHO1862, MHO11 and MHO33 of *M. hominis* using Unipro UGENE 48.1.
